# Graphene Oxide Increases Corneal Permeation of Ciprofloxacin Hydrochloride from Oleogels: A Study with Cocoa Butter-Based Oleogels

**DOI:** 10.3390/gels6040043

**Published:** 2020-11-23

**Authors:** Dilshad Qureshi, Barbiee Choudhary, Biswaranjan Mohanty, Preetam Sarkar, Arfat Anis, Miguel A. Cerqueira, Indranil Banerjee, Samarendra Maji, Kunal Pal

**Affiliations:** 1 Department of Biotechnology and Medical Engineering, National Institute of Technology, Rourkela 769008, India; dilshadq786@gmail.com; 2 Amity Institute of Biotechnology, Amity University, Noida 201301, India; barbieechoudhary13@gmail.com; 3 Institute of Pharmacy and Technology, Salipur 754202, India; biswaranjanm5@gmail.com; 4 Department of Food Process Engineering, National Institute of Technology, Rourkela 769008, India; preetamdt@gmail.com; 5 SABIC Polymer Research Center, Department of Chemical Engineering, King Saud University, Riyadh 11451, Saudi Arabia; aarfat@ksu.edu.sa; 6 International Iberian Nanotechnology Laboratory, Av. Mestre José Veiga s/n, 4715-330 Braga, Portugal; miguel.cerqueira@inl.int; 7 Department of Bioscience and Bioengineering, Indian Institute of Technology, Jodhpur 600036, India; indraniliit@gmail.com; 8 Department of Chemistry, SRM Institute of Science and Technology, Kattankulathur 603203, India

**Keywords:** oleogels, rice bran oil, cocoa butter, graphene oxide, rabbit, ocular drug delivery

## Abstract

In this work, oleogels of cocoa butter (CB), rice bran oil (RBO), and graphene oxide (GO) were prepared. The prepared oleogels were subjected to various characterization techniques such as bright-field microscopy, X-ray diffraction (XRD), crystallization kinetics, differential scanning calorimetry (DSC), and mechanical studies. The influence of increasing GO content on the in vitro drug release and ex vivo corneal permeation of the model drug (ciprofloxacin HCl—CPH) from the oleogels was also investigated. Bright-field micrographs showed that increment in GO content reduced the size of the globular particles of CB. XRD analysis revealed that CB was crystallized in its β’ and β polymorphic forms in the oleogels, which was in agreement with thermal studies. The mechanical characterization demonstrated that the presence of GO improved the elastic nature and stress-bearing properties of the oleogels. Moreover, GO altered the crystallization kinetics of CB in the oleogels in a composition-dependent manner. The in vitro release of CPH from the oleogels occurred through either Fickian diffusion or fat network relaxation or a combination thereof. Furthermore, the inclusion of GO enhanced the ex vivo permeation of CPH molecules across the caprine cornea. Hence, we concluded that the prepared oleogels could be explored as potential delivery systems for ophthalmic applications.

## 1. Introduction

Oleogelation (or organogelation) can be defined as the process of transforming a liquid vegetable oil into a gel-like structured formulation. The oleogels fabricated through the oleogelation technique are generally a three-dimensional thermoreversible network of oleogelator molecules that entraps the liquid oil [1]. The oleogelators that are used for the structuring of the oils are broadly classified as low-molecular-weight oleogelators (LMOGs) (e.g., triacylglycerol derivatives, free fatty acids) and high-molecular-weight oleogelator (HMOGs) (e.g., polysaccharides, proteins, polymers). It has been reported that the properties of oleogels vary significantly, which is dependent upon the molecular architecture of the oleogelator network [2]. Over the years, oleogels have been utilized in diverse fields ranging from food (e.g., preparation of cookies, cakes, chocolates, meat products, and ice-cream) and cosmetics to pharmaceutical industries (e.g., topical, ocular, and oral drug delivery systems) [3,4,5,6,7,8,9,10,11,12]. However, an incredible increment in the popularity and use of oleogel-based systems as novel drug delivery devices have been witnessed ever since. As compared to the conventional drug delivery systems, oleogels are typically inexpensive, biocompatible, resistant to microbial contamination, and stable for longer periods [13]. Furthermore, oleogel matrices can be used as excellent carriers for both, hydrophilic or hydrophobic cargos [13]. The lipophilic nature of the oleogels assists the penetration of the drug molecules across the biological tissues [14]. Currently, oleogels have become an advanced approach for periodontal [14], nasal [15], topical [10], parenteral [16], and ocular [17] drug delivery applications. Unlike other fields, there has been a huge lack of research regarding the usage of oleogels for the controlled release of ocular therapeutics. Only a few authors, including our research group, have designed novel oleogel-based systems and explored their propriety for ophthalmic drug delivery applications [11,13,17].

Cocoa butter (CB) is a natural fat, which is extracted from the beans of the cocoa tree (*Theobroma cacao*) [18]. *Theobroma cacao* is cultivated in Ivory Coast, Nigeria, Ghana, Brazil, Cameroon, Indonesia, Malaysia, and Ecuador [19]. The compositional profile of the triacylglycerols (TAGs) of CB considerably varies with the origin of the plant, season of collection of the beans, variety of the tree, and handling techniques during the extraction process [20]. Commonly found triacylglycerols (TAGs) in CB include glycerol-1-palmitate-2-oleate-3-stearate (POSt; P-palmitic, St-stearic, O-oleic; 33.7–40.5%), glycerol-1,3-distearate-2-oleate (StOSt; 23.8–31.2%), and glycerol-1,3-dipalmitate-2-oleate (POP; 13.6–15.5%) [21]. Further, CB also consists of three major fatty acids, namely, stearic (33.0–37.5%), oleic (32.5–36.5%), and palmitic acids (24.0–28.0%) [22]. The composition of the TAGs and fatty acids in CB affect the solid fat content, melting characteristics, and polymorphism of CB. At 20 °C, CB remains mainly as solid (~70% solid fat content) and melts in the temperature range of 30–35 °C [23]. This property of CB has been tremendously used in various industries, such as pharmaceutical (as topical and suppository bases), cosmetics (as lipsticks, soaps, moisturizers, perfumes, etc.), and food (in chocolate formulations) [24,25]. CB has been extensively used for the preparation of vaginal suppositories for the treatment of vaginal infections [26]. The solid lipid nanoparticles (SLNs) prepared using CB has been proposed for the targeted delivery of melanotransferrin antibody to treat brain cancer malignancies [27]. Morgan and coworkers (2016) patented an ocular formulation consisting of CB, shea butter, jojoba wax, and apricot kernel oil. The ocular comfort formulations have been proposed for the treatment of various ocular diseases, including dry eye syndrome, inflammation, fungal, and bacterial infection [28].

Rice bran oil (RBO) has shown excellent health-benefiting properties. Due to this reason, it has been widely used in food (as a salad oil and cooking oil), cosmetics (in skin-lightening and anti-aging products, sunscreen formulations, etc.), and pharmaceutical industries (as an anti-hyperglycemic agent, food stabilizer, etc.) [29,30,31,32]. RBO is obtained as a by-product of the rice milling process. It contains high amounts of vitamin E complex, polyphenols, tocotrienols, tocopherols, phytosterols, squalene, and polyunsaturated fatty acids (PUFA) [33]. It also contains γ-oryzanol, which is a natural antioxidant. γ-oryzanol is reported to confer many health benefits, including lowering of atherosclerosis, lowering of plasma cholesterol, inhibition of platelet aggregation, and lowering of fecal bile excretion [34]. RBO-based nanoemulsions have been explored as carriers for the oral delivery of active pharmaceutical ingredient (e.g., itraconazole) for the treatment of fungal infections [35]. Lipid-core nanocapsules (LCNs) of RBO have been explored for the treatment of various cutaneous diseases like leukemia, contact dermatitis, and tumors. The authors further reported that RBO could provide a protective effect against skin injuries that are induced by UVB radiation [36]. RBO has also been explored for the preparation of ocular ointments. The ointments could successfully re-hydrate the ocular tissues and could relieve the symptoms of dry eye [37].

Graphene oxide (GO) can be described as a single-layer sheet of graphite oxide. It is synthesized by exfoliating the graphite oxide in the form of layered sheets via mechanical stirring or sonication [38]. It exhibits some incredible properties, including fluorescence-quenching, an affinity for the aromatic ring, the capability to maintain high dispersibility in the aqueous medium, and electrical properties. These properties of GO make it a unique material [39]. Further, GO has been used as a reinforcing agent to impart mechanical stability to polymer matrices [40]. GO is used in various applications, namely, energy storage, biosensing, transparent electrode, cell scaffold, catalysis, and drug delivery. Due to the availability of a large surface area on both sides of the GO wafers, GO has been found to accommodate drug molecules, e.g., paclitaxel, camptothecin, and doxorubicin, within its structure [41]. Nano delivery systems of GO have been proposed for the development of the targeted drug delivery systems [42]. Since the nano delivery systems can significantly increase the drug loading capacity, the side effects due to repeated drug administration can be markedly reduced. Yan et al. (2012) found that targeted drug delivery in the eye could impart maximum medical benefits without affecting other organs. The authors studied the intraocular biocompatibility and toxicity of GO on human retinal pigment epithelium (RPE) cells. The intra-vitreal administration of GO did not change the eyesight, appearance, electroretinogram measurements, intraocular pressure, and histopathology of the rabbit eyes [43].

In the present study, we report the development of CB (oleogelator) and RBO (liquid phase)-based oleogels loaded with GO. The prepared oleogels were thoroughly evaluated for their microstructure, molecular interactions, crystallization kinetics, thermal, and mechanical characteristics. Additionally, the CB/RBO/GO-based oleogels were examined for their suitability as ocular drug delivery systems. For the purpose, CPH was used as an antimicrobial drug. The in vitro release and ex vivo corneal permeation of the CPH from the oleogels were also investigated in-depth. The non-irritant nature of the oleogels for ocular drug delivery applications was assessed by in vivo eye irritation test using rabbit models. Finally, the antibacterial activity of the pristine and CPH-oleogels was also investigated against model organisms.

## 2. Results and Discussion

### 2.1. Preparation of the Oleogels

**Figure 1:**
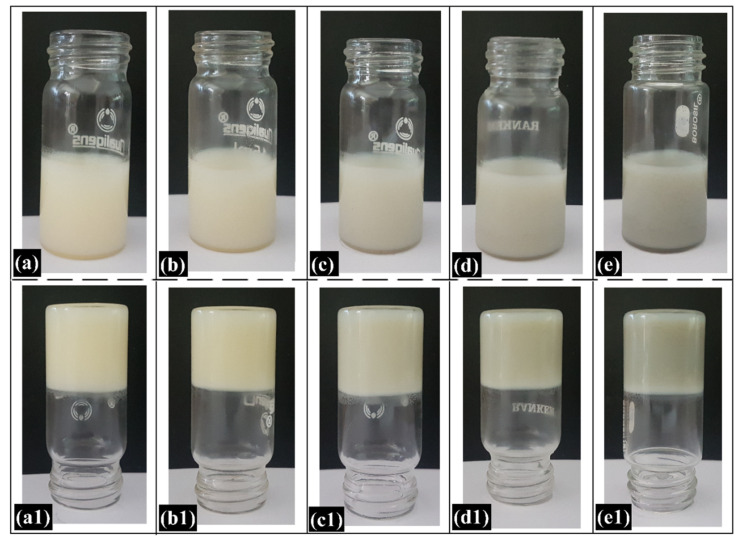
Pictures of the prepared oleogels: (**a**) CR1, (**b**) CR2, (**c**) CR3, (**d**) CR4, and (**e**) CR5; invert tube test of the oleogels: (**a1**) CR1, (**b1**) CR2, (**c1**) CR3, (**d1**) CR4, and (**e1**) CR5.

The critical gelator concentration (CGC) of CB in RBO was found to be 35% (*w*/*w*). At 45 °C, the mixture of CB and RBO appeared yellow translucent liquid. As the mixture was cooled down to 25 °C, it transformed into an opaque and semi-solid formulation (Figure 1). The inverted tube method was used to confirm the formation of the oleogels. It was observed that the inversion of the tube did not induce the downward flow movement of the formulations. This is indicative of the fact that the RBO was immobilized entirely within the fat network of CB and therefore formed a gelled structure. Further, the incorporation of GO in different concentrations did not affect the formation of the oleogels. The prepared oleogels were found to be opaque, smooth, and semi-solid in appearance. However, they differ in terms of their color. The addition of GO resulted in the formation of black-colored oleogels, whose intensity varied with the GO content. Differences in the color of the oleogels were associated with the incorporation of GO in different concentrations. Dao et al. (2015) have also found that the inclusion of GO into stearic acid latex resulted in the formation of the black-colored composites [44]. Additionally, the addition of CPH (model drug) into the formulations did not affect the gelation process of the oleogels.

### 2.2. Bright-Field Microscopy Analysis

**Figure 2:**
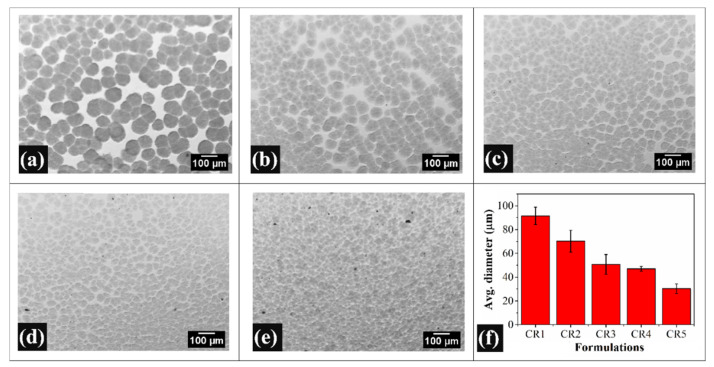
Bright-field micrographs of the oleogels: (**a**) CR1, (**b**) CR2, (**c**) CR3, (**d**) CR4, (**e**) CR5, (**f**) Diameter of crystallized fat particles (mean ± standard deviation).

The bright-field micrographs of the oleogels showed the presence of conjoined globular particles in a homogeneous matrix system (Figure 2). The size of the globular particles was bigger within the control formulation (CR1). These particles were made up of solid fats that are present in the CB [45]. The network of the conjoined globular particles of CB was responsible for immobilizing the RBO (liquid fat phase). The micrographs of the composite oleogels demonstrated tiny black particles dispersed throughout the matrix, which was recognized as GO. Additionally, it is evident that an increment in GO concentration (CR2–CR5) correspondingly increased the number of these black particles within the oleogel matrices. Interestingly, in CR4 and CR5, GO sheets showed aggregation (large size black particles). The aggregation of GO could be accounted to the hydrophilic interactions among the GO sheets, which were dispersed within the highly lipophilic oleogel network. It was observed that the addition of GO to the oleogel decreased the size of the globular particles. A significant reduction in the size of these particles was observed as the concentration of the GO was increased. This suggests that the size and the growth of the fat particles were dependent on the concentration of GO.

The average particle size of the solid fats of each sample was calculated using ImageJ software. The particle size analysis showed that there was a monotonous decrease in the average size of the fat particles as the GO content in the formulations was increased. The results suggest that the addition of GO does not promote the formation of larger fat crystals. This can be accounted to the fact that GO may be acting as nucleation points, which may be helping in altering the growth of the fat crystals. Liu et al. (2014) have previously reported that an increase in the GO content increases the formation of nucleating points [46]. These increased nucleation points helped in the homogeneous crystallization of the solid fats throughout the warm molten mixture of CB/RBO/GO and can explain the formation of the increasingly smaller fat crystals.

### 2.3. FTIR Analysis

**Figure 3:**
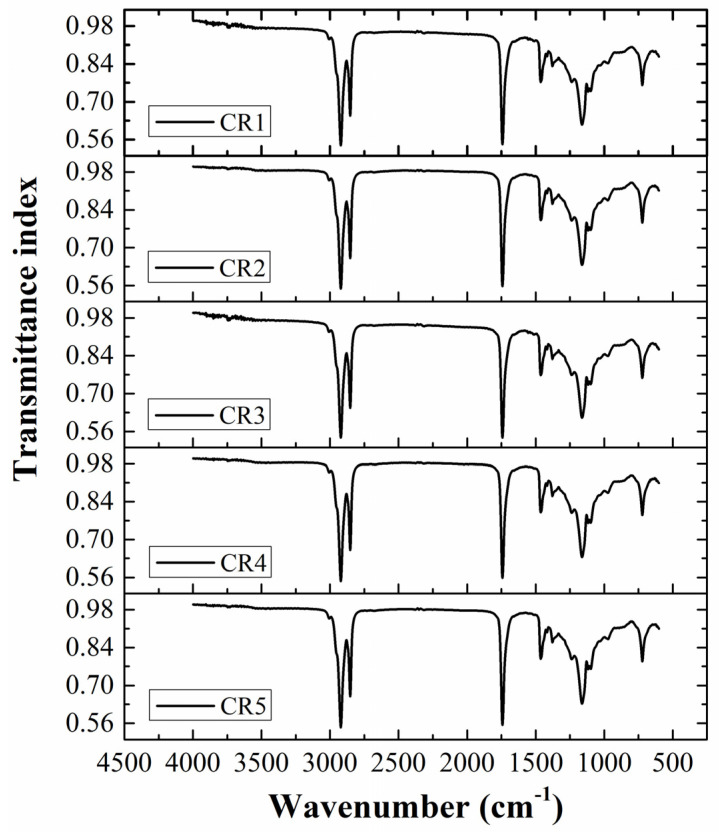
FTIR spectra of the prepared oleogels.

The chemical interactions within the CR1 (control formulation) and GO-containing oleogels (CR2-CR5) were analyzed by FTIR spectroscopy in the spectral range of 4000–600 cm^−1^ (Figure 3). The FTIR spectra obtained for all the prepared oleogels showed characteristic peaks of CB and RBO. The control oleogel (CR1) showed a sharp signal at 3005 cm^−1^ in the spectrum, which can be attributed to the stretching vibration of cis olefinic double bonds (=C–H) [47]. The peaks obtained at 2922 cm^−1^ and 2852 cm^−1^ were due to the asymmetrical and symmetrical stretching vibration of methylene (–CH_2_) groups in the fatty acid molecules [47]. The carbonyl (–C=O) stretching vibration of the ester bond was evident from the peak observed at 1742 cm^−1^ [48]. Two distinct peaks noticed at 1463 cm^−1^ and 1377 cm^−1^ can be associated with the bending vibration of methylene (–CH_2_) and methyl (–CH_3_) groups [48]. The vibrational signals observed at 1236 cm^−1^, 1161 cm^−1^, and 1116 cm^−1^ can be assigned to different stretching vibrations of the C–O group in esters. The peak at 1236 cm^−1^ was due to the stretching vibration of C–O in –C(=O)–CH_2_ of ester moiety. A sharp and strong band recorded at 1161 cm^−1^ can be assigned to the C–O bond stretching vibration in HC–O–(C=O) of the ester group. However, the weak vibrational signal at 1116 cm^−1^ corresponded to the C–O stretching in O–C–C of the ester [49]. A medium-strong vibrational signal corresponding to the stretching of C–C bonds present in the hydrocarbon chains was noticed at 1097 cm^−1^. Additionally, a very sharp peak observed at 722 cm^−1^ can be associated with the methylene group rocking vibration [49].

Interestingly, in the case of GO-containing oleogels, the position of the above-mentioned major peaks was observed to be identical. This indicated that the intermolecular interactions among the components of the oleogels were either absent or quite weak. Mehrali and coworkers (2013) also reported the absence of significant interactions among the functional groups of palmitic acid and GO [50]. Furthermore, the characteristic GO vibrational peaks reported by Vuppaladadium et al. (2020) such as 3580 cm^−1^ (O–H stretching), 1624 cm^−1^ (C=C aromatic ring stretching), 1399 cm^−1^ (C–O carboxyl), 1223 cm^−1^ (C–O–C epoxy stretching), and 1060 cm^−1^ (C–O alkoxy), could not be observed in the spectra of the CB/RBO/GO-based oleogels [51]. This can be explained by the presence of GO in meager concentration. CR5 contained a maximum concentration of GO, which was 0.05% (wt%) of the formulation. In the said concentration range, though the physical process of crystallization was affected (as evident from the microstructure), no signs of chemical interactions were observed in the FTIR analysis.

### 2.4. XRD Analysis

**Figure 4:**
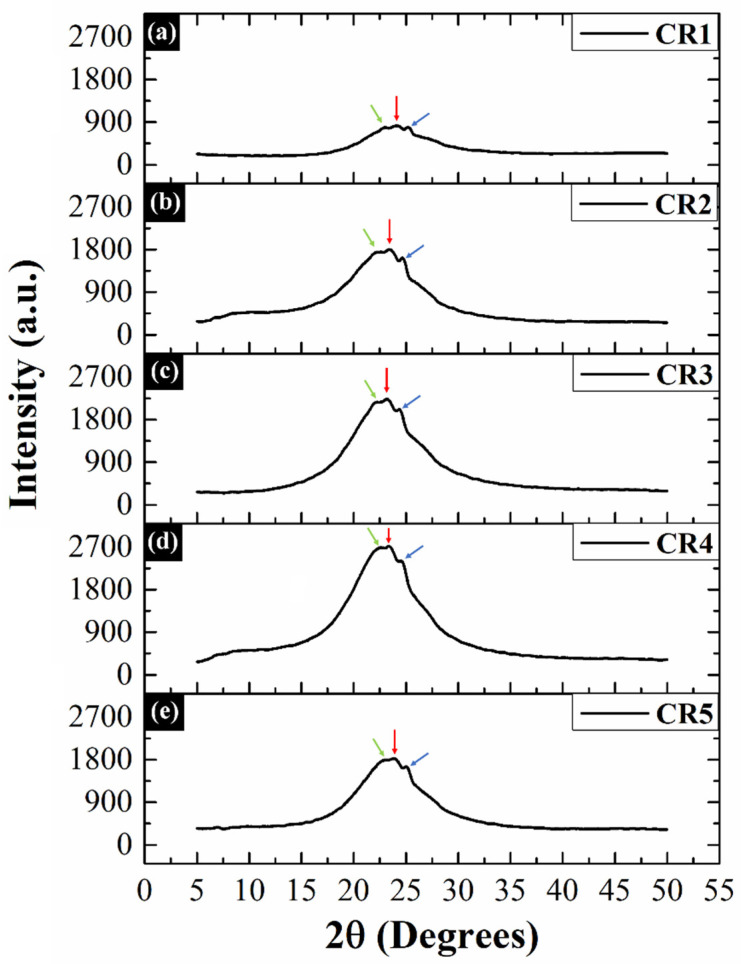
X-ray diffraction patterns of the prepared oleogels: (**a**) CR1, (**b**) CR2, (**c**) CR3, (**d**) CR4 and (**e**) CR5.

Figure 4 depicts the XRD profiles of the prepared oleogels. The diffractogram of the control oleogel (CR1) showed three Bragg peaks at 23.02° 2θ (weak; green arrow), 24.06° 2θ (strong; red arrow), and 25.22° 2θ (medium strong; blue arrow) (Figure 4). These short spacing peaks corresponded to the d-spacing of 4.48 Å, 4.29 Å, and 4.10 Å (Bragg’s law; Equation (1)). However, these diffraction peaks shifted their position in GO-containing oleogels (CR2-CR5). The weak shoulder peak observed at 23.02° 2θ in CR1 shifted its position to lower diffraction angles in CR2 (22.7° 2θ; 4.55 Å), CR3 (22.24° 2θ; 4.64 Å), CR4 (22.58° 2θ; 4.57 Å), and CR5 (22.92° 2θ; 4.50 Å). The major strong peak (24.06° 2θ) was also recorded at lower diffraction angles of 23.34° 2θ (CR2; 4.42 Å), 23.16° 2θ (CR3; 4.46 Å), 23.36° 2θ (CR4; 4.42 Å), and 23.86° 2θ (CR5; 4.33 Å). Similarly, the third peak also shifted to the lower positions in CR2 (24.64° 2θ; 4.19 Å), CR3 (24.36° 2θ; 4.24 Å), CR4 (24.54° 2θ; 4.21 Å), and CR5 (25.00° 2θ; 4.13 Å) as compared to the CR1. Interestingly, the XRD patterns of the GO-containing oleogels did not display the characteristic Bragg peak (~10° 2θ) of GO, which has been well-reported in previous literature [51,52]. This can be owed to the sparse amount of GO, which was used for the preparation of composite oleogels. A gradual increase in the intensity of these three peaks was observed as GO concentration was increased till CR4. Thereafter, a sudden decrease in the same was observed in CR5.

The diffraction spectra of the oleogels were deconvoluted to extract the information regarding the crystalline and amorphous nature of the prepared formulations (Figure S1). The deconvolution of the peaks using the non-linear peak fitting method could resolve the XRD spectra in a total of eight peaks (Table S1). However, the three diffraction peaks (blue, cyan, and magenta) obtained at the Bragg angle positions approximately similar to that of the major peaks (previously mentioned) were used for further analysis. Interestingly, no diffraction peak corresponding to GO could be resolved through deconvolution. The peak position and full width at half maximum (FWHM) values of the deconvoluted peaks have been tabulated in Table 1. The variations in the FWHM of diffraction peaks can be correlated to the crystalline nature of the oleogels. The average FWHM value of the prepared oleogels increased monotonously from CR1 (2.122) to CR3 (3.228). This indicated that the introduction of GO in lower wt% increased the amorphous nature of the crystal network in CR2 and CR3. As the concentration of GO was further increased, the average FWHM value correspondingly decreased in CR4 (2.216) and CR5 (1.969). The lowest average FWHM of CR5 suggested that the CB crystallites in this formulation were comparatively more crystalline than the remaining oleogels. The interplanar spacing (d-spacing) in the lattice structure of the prepared oleogels was calculated using Bragg’s equation (Equation (1); Table 1) [53]. The parameter d-spacing provides information regarding the lattice compactness [54]. The average d-spacing value of CR1 was 4.112 Å. As the content of GO increased in the oleogels, the average d-spacing value gradually decreased from CR2 to CR5. Amongst all the GO-containing oleogels, CR5 exhibited the lowest average d-spacing value (4.275 Å), which might be due to the formation of a compact lattice structure of the crystalline domains [55]. Hence, it can be speculated that the addition of GO altered the compactness of the arrangement of the CB fat crystals by manipulating the crystallization of the CB. An analysis of all the deconvoluted peaks (Table S1) suggested that the formulations showed peaks at ~4.13 Å, ~4.20 Å, and ~4.32 Å. This is suggestive of the formation of β’ (either Form III or Form IV) polymorphs [56]. Additionally, another peak at ~4.58 Å was observed. This indicated the possibility of the presence of β (Form V or Form VI) polymorphs, too [56].

Next, the crystallite size (D size) was calculated using the Debye–Scherrer equation (Equation (2); Table 1) [57]. We observed that the CR1 exhibited the average crystallite size of 9.010 nm, while the same dropped drastically in CR2 (4.760 nm) as GO was added in as low as 0.005 wt%. A further increment in the GO concentration monotonously elevated the average size of the crystallites in the crystal network of CR3 (5.670 nm), CR4 (10.090 nm), and CR5 (11.433 nm). The average size of the crystallites was the largest in CR5, which suggested that the formation of highly compact and crystalline CB crystals. The average lattice strain of the fat crystallite domains was also calculated (Equation (3); Table 1). Risan et al. (2018) have reported that nanoparticles can alter the nucleation process and particle assembly. This can result in the alteration of the anisotropy and lattice strain of the crystals [58]. In our study, we found that the average lattice strain value of CR1 (0.041) was substantially lower than that of GO-containing oleogels, viz, CR2 (0.063) and CR3 (0.071). The higher values of the lattice strain indicated a reduction in the crystallinity of these oleogels. It was observed that the average lattice strain reached its critical higher limit in CR3. Thereafter, there was a decrease in the average lattice strain in CR4 and CR5, which showed similar average lattice strain values as that of CR1 (0.041). Since the lattice strain is inversely related to the crystal perfectness, low average lattice strain values indicated that the fat crystal network of CR4 and CR5 was highly ordered [59]. In a nutshell, we observed that the inclusion of GO in the oleogels enhanced the compactness and formation of large-sized crystallites with very low lattice imperfections. Additionally, amongst the prepared oleogels, we speculate that CR5 is an optimum composition in terms of compact crystal structure and high crystallinity. This may significantly affect all the physical and thermal properties of CR5 as compared to the remaining oleogels.

(1) 2dsinθ=nλ

where *d* is the interplanar placing of the crystal, *θ* is the diffraction angle, *n* is an integer, and *λ* is the wavelength of the incident X-rays (1.789 Å).

(2) $$D=\frac{k\lambda }{\beta \;\cos\theta }$$

where *D* is the crystallite size, *k* is the shape factor or Scherrer constant (=0.89, due to unknown crystal shape), *β* is the FWHM of the diffraction peak in radians, and *λ* is the wavelength of the X-rays used (1.789 Å).

(3) $$\varepsilon =\frac{\beta }{4\tan\theta }$$

where *ε* represents the strain of the material, *β* is the FWHM, and *θ* represents Bragg’s angle.

### 2.5. Thermal Analysis

#### 2.5.1. Crystallization Kinetics

**Figure 5:**
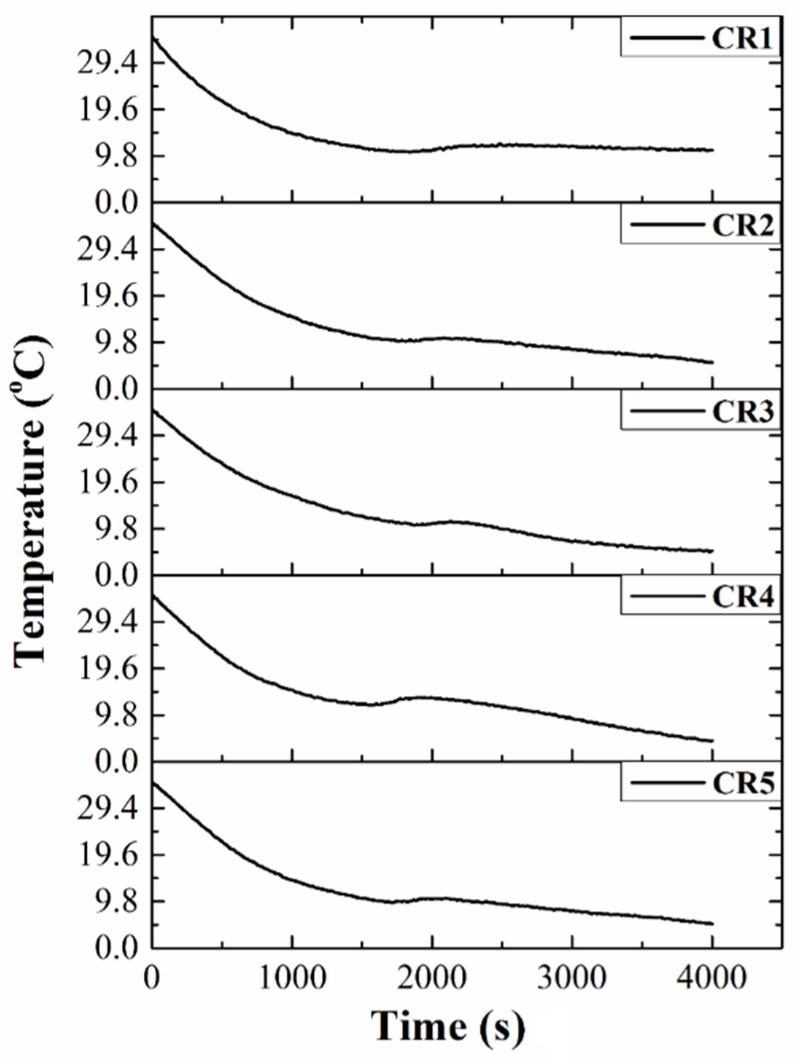
Crystallization profiles of the prepared oleogels at 5 °C.

The crystallization profiles showed an initial decrease in the temperature as the time progressed (Figure 5). Then, at a particular temperature, the temperature profiles remained nearly constant (isothermal phase) for a definite duration. Similar results have been reported by Uvanesh et al. (2016). The group reported that the crystallization of the fat molecules occurs under isothermal conditions [60]. Under such crystallization conditions, the solid and the liquid components of the formulation tend to remain in a local equilibrium [61]. After the isothermal phase, the temperature profiles again started to decrease. To enhance our understanding of the kinetics of crystallization, we have fitted the initial part of the temperature profile to the exponential equation (Equation (4); Figure S2) [62]. The parameters of the crystallization kinetics have been summarized in Table 2. *k* is the rate of crystallization and is dependent on the nucleation, temperature, and crystal growth conditions [63]. The negative value of the *k* was due to the decaying exponential nature of the crystallization profiles. CR1 exhibited the highest rate of crystallization (−0.95 °C/m·s) among all formulations. As compared to CR1, the rate of crystallization decreased in all GO-containing oleogels. The rate of crystallization was similar in the case of CR2 (−0.82 °C/m·s), CR4 (−0.89 °C/m·s), and CR5 (−0.87 °C/m·s). However, the rate of crystallization was lowest in CR3 (−0.74 °C/m·s). The alterations in the crystallization rate of the CB can be explained by the amphiphilic nature of GO. It has been reported that GO sheets are amphiphilic materials in which the hydrophilic and hydrophobic domains are distributed in an edge-to-center fashion. The amphiphilic nature is typically a property of long-chain surfactants [64]. Hence, it can be speculated that GO might have acted as an emulsifier-based additive during the crystallization process. As per the literature, the dissimilarities in the structure of the emulsifier (here GO) and TAGs can hinder the process of crystallization [65]. This could explain the delay in the crystallization of CB in GO-containing oleogels. The structural dissimilarity between the sheet structure of GO and long-chain TAGs in CB might have entailed a delay in the nucleation, which indicated the decrement in the crystallization rate of CB in CB/RBO/GO-based oleogels.

(4) $$y\left(t\right)=a.e^{kt}$$

where *y*(*t*) = temperature of the formulation with respect to time (*t*), *a* is the initial temperature of the sample (°C), *t* is the time (s) and *k* is the rate of crystallization.

#### 2.5.2. DSC Analysis

**Figure 6:**
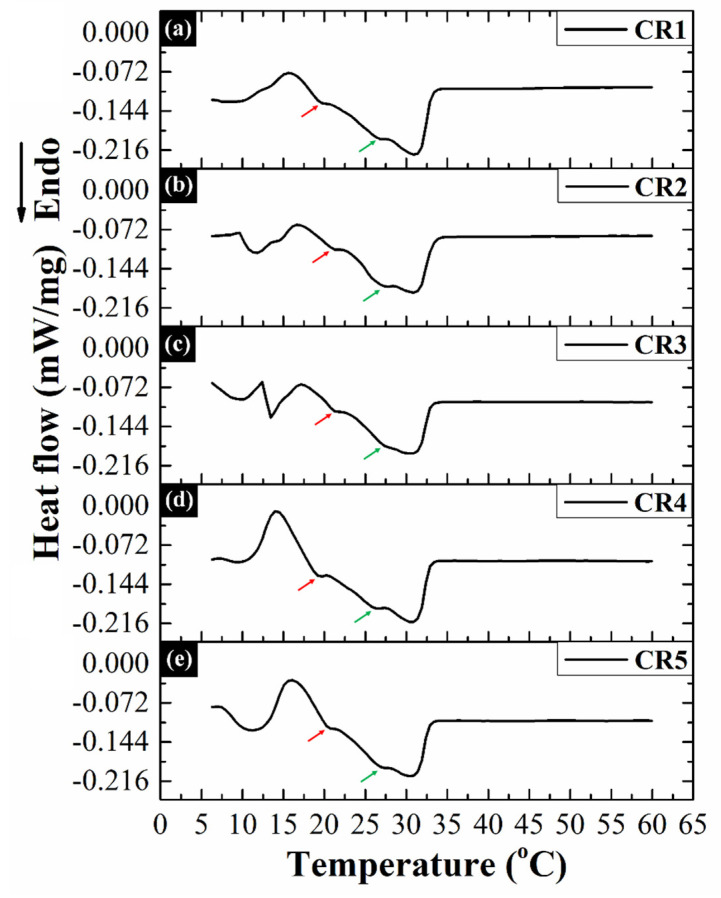
DSC thermograms of the prepared oleogels: (**a**) CR1, (**b**) CR2, (**c**) CR3, (**d**) CR4, and (**e**) CR5.

The thermograms of the prepared oleogels showed a prominent broad endothermic signal at ~31°C (Figure 6). This endothermic signal indicated the presence of the most stable β (Form V)-polymorph of CB [56,66]. A careful observation of the thermograms revealed that the endothermic peaks showed two distinct shoulder peaks, which have been indicated by red and green arrows (Figure 6). The respective positions of these additional peaks have been tabulated in Table 3. To have a better understanding of the melting endotherms, we deconvoluted the major endothermic peak. The non-linear peak fitting analysis resolved this peak into three characteristic peaks, which were obtained at ~20, ~28, and ~31 °C (Figure S3). The endothermic signal observed at ~20 °C can be attributed to the α-(Form II) polymorph of CB [56]. The position of another endothermic peak (~28 °C) matched with the published temperature range (27–29 °C) for β’ (Form IV) polymorphic form of CB [56,66,67]. Moreover, the third peak (~31 °C) confirmed the presence of β (Form V) polymorph of the CB crystals [66]. Furthermore, the area under the peaks associated with β’ and β polymorphs of the CB was compared. It was observed that the proportion of β’ polymorphic form in the crystal network of the prepared oleogels was more prominent as compared to the β form. The presence of β-polymorph is desired in products due to its appropriate melting point. The products containing β-polymorph melts in the mouth very rapidly [68].

### 2.6. Mechanical Analysis

**Figure 7:**
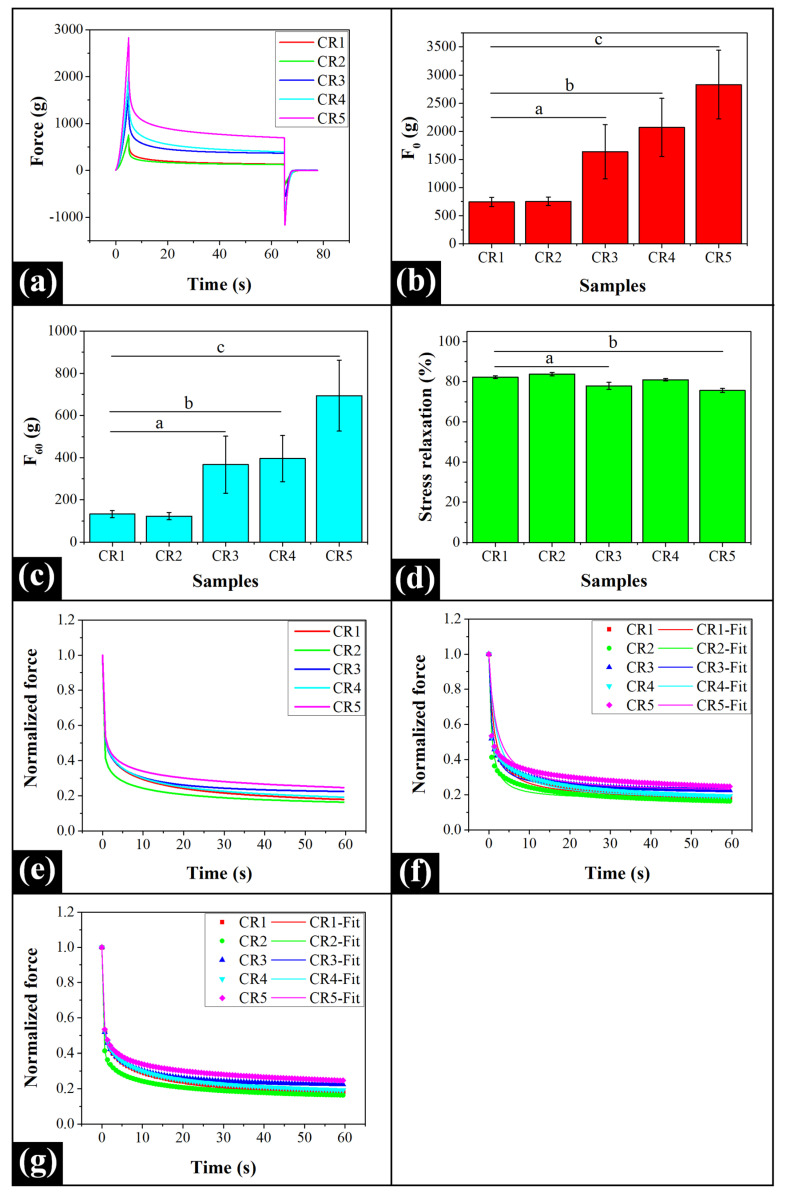
Mechanical analysis of the prepared oleogels: (**a**) stress relaxation (SR) profiles, (**b**) *F*_0_ maximum force (mean ± standard deviation) (**c**) *F*_60_ residual force (mean ± standard deviation), (**d**) %SR (mean ± standard deviation), (**e**) normalized relaxation profiles, (**f**) Peleg’s model fitting, and (**g**) Wiechert model fitting. Different lowercase letters denote significant differences at *p* < 0.05 level.

A deeper understanding of the viscoelastic and the mechanical characteristics of the prepared oleogels was achieved by performing stress relaxation (SR) studies [69]. The SR profiles have been provided in Figure 7a while the stress relaxation parameters have been tabulated in Table S2. The visual observation of the SR profiles suggested a similar-natured profile for all the oleogels. In this study, the samples were subjected to uniaxial compression using a cone probe, and the strain was maintained for 60 s. The maximum force (*F*_0_) sensed by the load cell when the probe reached the target distance (5 mm) signifies the firmness of the formulations [70]. It can be noticed that, as compared to CR1, the firmness of the GO-containing oleogels was significantly higher (*p* < 0.05), except for CR2 (*p* > 0.05) (Figure 7b). The rise in GO concentration monotonously increased the firmness of CR3, CR4, and CR5 in contrast to CR1 and CR2 (*p* < 0.05). This increased firmness might be due to the reinforcing effect exerted by the GO particles [40]. However, the firmness of the oleogel network structure was similar in the case of CR3, CR4, and CR5 (*p* > 0.05). The force sensed by the cell at the end of the relaxation process (60 s) can be termed as “residual force” (*F*_60_) [71]. Uvanesh et al. (2016) have reported that higher values of *F*_60_ (residual force) signify the formation of a more stable network of fat architecture [72]. The variation in the residual force values of CR1 and CR2 were non-significant (*p* > 0.05) (Figure 7c). However, the said formulations exhibited significantly lower *F*_60_ values in comparison to CR3, CR4, and CR5 (*p* < 0.05). An increment in GO content imparted no significant variations in the *F*_60_ values of CR3 and CR4 (*p* > 0.05). However, the residual force was comparatively higher in CR5 as compared to CR3 and CR4 (*p* < 0.05). It is worth noticing that the *F*_60_ parameter signifies the residual elastic component of the crystal network of the formulations. Hence, it can be concluded that the addition of GO helped in the formation of a more stable structure in the formulations that had higher levels of GO. Sagiri et al. (2015) have mentioned that %SR is the measure of the molecular arrangement of all the constituents that are present in the gel [73]. Hence, the %SR was calculated to study the percentage decrease in the force values from the maximum to the end of the relaxation time (Equation (5)). We noticed that the %SR value of CR1 did not significantly vary from that of CR2 and CR4 (*p* < 0.05) (Figure 7d). Conversely, the %SR values of CR3 and CR5 were significantly lower than CR1 (*p* < 0.05). In the case of GO-containing oleogels, CR2 exhibited significantly higher %SR as compared to CR3, CR4, and CR5 (*p* < 0.05). It was observed that the strained crystal network of CR3 significantly relaxed to a lower extent as compared to CR4 (*p* < 0.05). However, the extent of relaxation was not statistically significant in comparison to CR5 (*p* > 0.05). The inclusion of GO at the highest concentration reduced the deformation in CR5 as compared to CR4 (*p* < 0.05). The observations suggested that the addition of GO under the reported levels increased the firmness and elastic behavior of the oleogels.

In an attempt to evaluate the viscoelastic properties of the oleogels in-depth, the relaxation profiles of the oleogels were further analyzed by fitting the data using modified Peleg’s equation (Equation (6)) [74]. For this purpose, the normalized SR data were plotted against time (Figure 7e) and were fitted to have a good correlation between the experimental and the fitted data (Figure 7f; Table S2). A correlation coefficient of >0.95 was accepted as a good fit (Table S2). *k*_1_ is the initial rate of relaxation. The incorporation of GO in as low as 0.005 wt% concentration reduced the *k*_1_ value of CR2 (0.685) as compared to CR1 (1.455). This observation suggested that the amorphous crystal network of CR2 relaxed more quickly in contrast to the crystalline network of CR1 (as observed from XRD) under low-stress conditions. Thereafter, a further increment in GO content correspondingly increased the *k*_1_ values in CR3 (0.925), CR4 (2.060), and CR5 (2.254). As expected, the *k*_1_ values were considerably higher in CR4 and CR5 as compared to the remaining oleogels. These higher *k*_1_ values indicated the formation of an elastic and more rigid fat crystal network in these formulations. The other parameter (*k*_2_) denotes the extent of relaxation. The values of *k*_2_ were found to be similar in all the formulations.

Peleg’s model is an empirical mathematical model to analyze stress relaxation, so we have further analyzed the SR profile using the Wiechert model (Equation (7), Figure 7g) [75]. In this model, *P*_0_, *P*_1,_ and *P*_2_ are the pre-exponential factors (Table S2). *P*_0_ values are the markers of the retained elastic component of the prepared oleogels, whereas, *P*_1_ and *P*_2_ are the initial and the delayed elastic components, respectively. We have found that the values of *P*_0_ followed the same pattern as k_2_ in all the formulations, except CR4. The values of *P*_1_ were in the order: CR1 > CR4 > CR3 > CR5 > CR2. The values of *P*_2_ were found to be approximately similar in all the prepared oleogels. *τ*_1_ is the instantaneous relaxation time. The *τ*_1_ values decreased until CR3, and thereafter, it increased in CR4 and CR5 (Table S2). This suggested an increase in the immediate relaxation process due to the molecular reorganization of the fat molecules when GO was incorporated even in lower amounts (CR2 and CR3). This observation satisfactorily concurred with our XRD results. A quick reorientation of the CB crystal network in the CR2 and CR3 can be accounted for the decrement in their respective average crystallite sizes. An increase in the GO content in CR4 and CR5 delayed the immediate relaxation process suggesting that there might be an increase in the rigidity of the fat network. Hence, the easy molecular rearrangement was not possible. *τ*_2_ denotes the delayed relaxation time, and the value of *τ*_2_ was highest in CR1 and lowest in CR2 and CR3 (Table S2). The lowest *τ*_2_ value in CR2 and CR3 may be explained by the higher average lattice strain values (i.e., higher lattice imperfections) in these formulations as compared to the rest of the oleogels. This might have resulted in the breakage of their crystal network structure under strain (Table 1). As the GO content was increased, there was a consequent increase in the delayed relaxation time. This observation suggested that CR1 had the most stable fat network, followed by CR5. Due to this reason, the fat crystal network of CR5 exhibited excellent elasticity and solid-like behavior.

(5) $$\%\;SR=\left(\frac{F_{0}-F_{60}}{F_{0}}\right)\times 100$$

where *F*_0_ is the maximum force applied using the cone probe at a distance of 5 mm, and *F*_60_ is the residual force at the end of the relaxation.

(6) $$\frac{(F_{0}-F\left(t\right))t}{F_{0}}=k_{1}+k_{2}\left(t\right)$$

where *F*_0_ is the maximum force applied using the cone probe at a distance of 5 mm, *F*(*t*) is the force decay as a function of time, *k*_1_ is the initial rate of relaxation, and *k*_2_ is the extent of relaxation.

(7) $$P_{t}=P_{0}+P_{1}.e^{-1/\tau_{1}}+P_{2}.e^{-1/\tau_{2}}$$

where *P*_0_, *P*_1_, and *P*_2_ are spring constants, and *τ*_1_ and *τ*_2_ are the time constants.

### 2.7. Drug Diffusion and Permeation Studies

**Figure 8:**
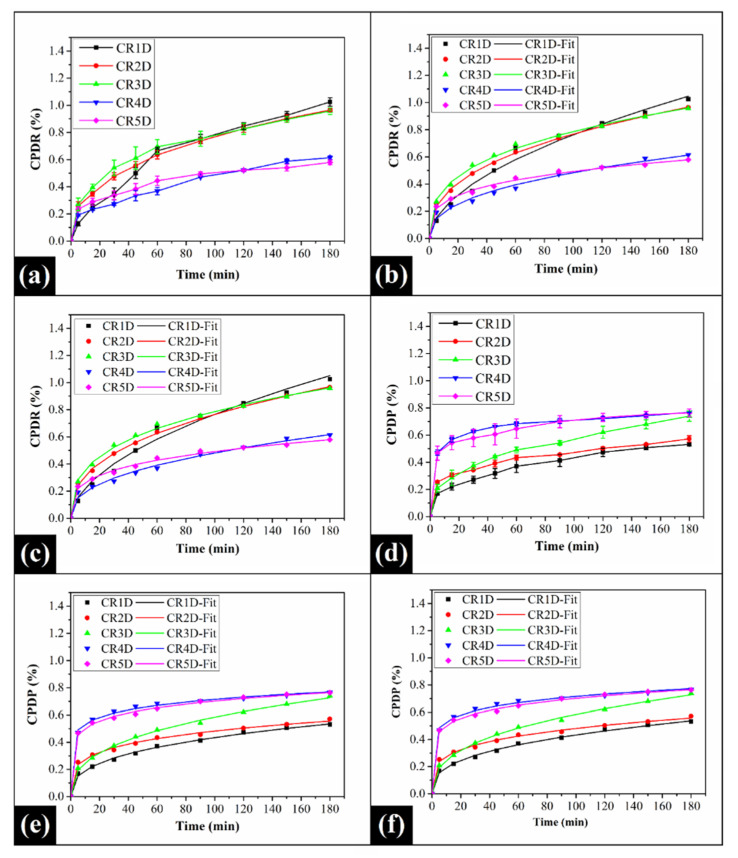
In vitro drug diffusion profiles of the prepared oleogels: (**a**) % cumulative percent drug release (CPDR), (**b**) Korsmeyer–Peppas model fitting of the CPDR profiles, (**c**) Peppas–Sahlin model fitting of the CPDR profiles; Ex vivo corneal permeation profiles: (**d**) % cumulative percent drug permeation (CPDP), (**e**) Korsmeyer–Peppas model fitting of the CPDP profiles, and (**f**) Peppas–Sahlin model fitting of the CPDP profiles.

The release profiles representing the diffusion of CPH (model drug) from the prepared oleogels has been shown in Figure 8. It was observed that the control oleogel (CR1D) demonstrated the highest cumulative percent drug release (CPDR) for 180 min amongst all the formulations (*p* < 0.05). The presence of GO could not significantly affect the CPDR of CPH in CR2D and CR3D (*p* > 0.05). However, as the concentration of GO gradually increased, the CPDR significantly decreased in CR4D to CR5D as compared to CR1D, CR2D, and CR3D (*p* < 0.05) (Figure 8a). The minimum release of CPH in CR5D can be explained by the entrapment of the drug molecules within the large CB crystallites (as observed from XRD analysis). Moreover, it has been already observed that the firmness (*F*_0_ value) of the said formulation was quite high as compared to the other formulations. This might have decreased the flexibility and relaxation of its crystal network structure, which in turn, reduced the release of CPH molecules. Further, to have a better understanding of the diffusion of the solute (drug) molecules, the release profiles were fitted to various drug release kinetics models. The fitting of the release kinetics models is performed for 60% of the fractional release [76]. In our case, the fractional release was much below the said limit. Hence, the full-length data was used for the modeling purpose. The release profiles were a good fit for both Korsmeyer–Peppas (KP) (Figure 8b) and Peppas–Sahlin (PS) (Figure 8c) model data (Equations (8) and (9), respectively) [77,78]. The model data parameters have been tabulated in Table 4. The release constant (K) indicates the diffusion of the drug molecules across the formulation matrices. The addition of GO in lower amounts increased K value in CR2D (0.127) and CR3D (0.167) in contrast to CR1D (0.064). However, a further increase in the GO content substantially decreased the K value in CR4D (0.076). Thereafter, the CPDR value of CR5D was again increased (0.142). The value of *n* of the KP model was <0.45 in all the GO-containing oleogels [79]. This suggests that the drug release occurred via Fickian diffusion. The observed value of *n* (i.e., <0.45) could be explained by the hydrophilic nature of CPH molecules, due to which it exhibited low-solubility in the prepared oleogels. Hence, the CPH was present in the particulate form in the drug-loaded oleogels. In this particular case, the release of the model drug might have been controlled by the penetration of water molecules in the formulations and consequent CPH dissolution [65,80]. On the contrary, the diffusion through CR1D occurred via non-Fickian diffusion (*n* > 0.45) [81]. However, this model is empirical, and to overcome the limitations of this model, the in vitro drug release process was further analyzed with the help of the PS model. As per the model parameters, the diffusion due to both Fickian release (*K_d_*) and fat network relaxation (*K_r_*) played essential roles in the in vitro drug release process. To increase our understanding, the ratio of the diffusion due to Fickian release and diffusion due to relaxation (*K_d_*/*K_r_*) was calculated. The result suggested that diffusion due to the relaxation of fat chains was predominant in CR1D and CR3D. In CR4D, the diffusion due to Fickian release was predominant. However, in CR2D and CR5D, the drug release was due to the similar contributions from Fickian diffusion and fat network relaxation process.

The permeation profile of the drug molecules through the goat cornea has been shown in Figure 8d. The cumulative percent drug permeation (CPDP) across the excised goat cornea showed a monotonous increment from CR1D until CR4D, where a critical higher level was reached. Thereafter, it remained constant in CR5D. Interestingly, the amount of drug permeated across the cornea was very less as compared to the drug transported across the dialysis membrane in the diffusion study. The differential results in the drug release and drug permeation studies can be accounted to the fact that in the CPDP, the properties of the cornea (a biological tissue) play an important role in the drug transportation process. In general, the cornea is a protective tissue, which prevents diffusion of the external molecules within the internal structures of the eye, thereby preventing the internal structures of the eye from damage. Further, the oleogel formulations must also have interacted with the corneal tissue differently. The results suggest that the incorporation of GO within the oleogels has improved the corneal permeation of the drug. To enhance our understanding of the drug transport process across the cornea, we analyzed the corneal permeation using the KP and PS drug release kinetic models (Figure 8e,f, respectively). The values of the release constant varied in a manner similar to that observed in the drug diffusion study. Moreover, the *n* value of the KP model was found to be less than 0.45 in all the formulations (Table 4). This suggested that the transport across the corneal tissue was Fickian-diffusion mediated. Further, the permeation profile was fitted to the PS model, and the ratio of the permeation due to Fickian release and permeation due to relaxation (*K_d_*/*K_r_*) was calculated (Table 4). Herein, we have found that the Fickian diffusion played a significant role in the transportation of the CPH molecules across the corneal tissue in all the prepared formulations, except in CR3D, where the permeation due to polymer (corneal tissue) relaxation process played a significant role in the drug transportation process. We have also observed that the *K_d_*/*K_r_* value in CR4D was unexpectedly high. This may be explained by the negligible polymer chain relaxation within the fat crystal network of the said formulation.

(8) $$F=\left(\frac{M_{t}}{M}\right)=K.t^{n}$$

where $\frac{M_{t}}{M}$ is the fraction of drug accumulated in the solution at time *t*, *K* is the release rate constant, and *n* is the slope.

(9) $$\left(\frac{M_{t}}{M_{o}}\right)=K_{d}.t^{m}+K_{r}.t^{2m}$$

where $\frac{M_{t}}{M_{o}}$ is the fraction of drug accumulated in the solution at time *t*, *K_d_* is the diffusion due to Fickian release, *K_r_* is the diffusion due to relaxation, and *m* is the slope.

### 2.8. Antimicrobial Analysis

**Figure 9:**
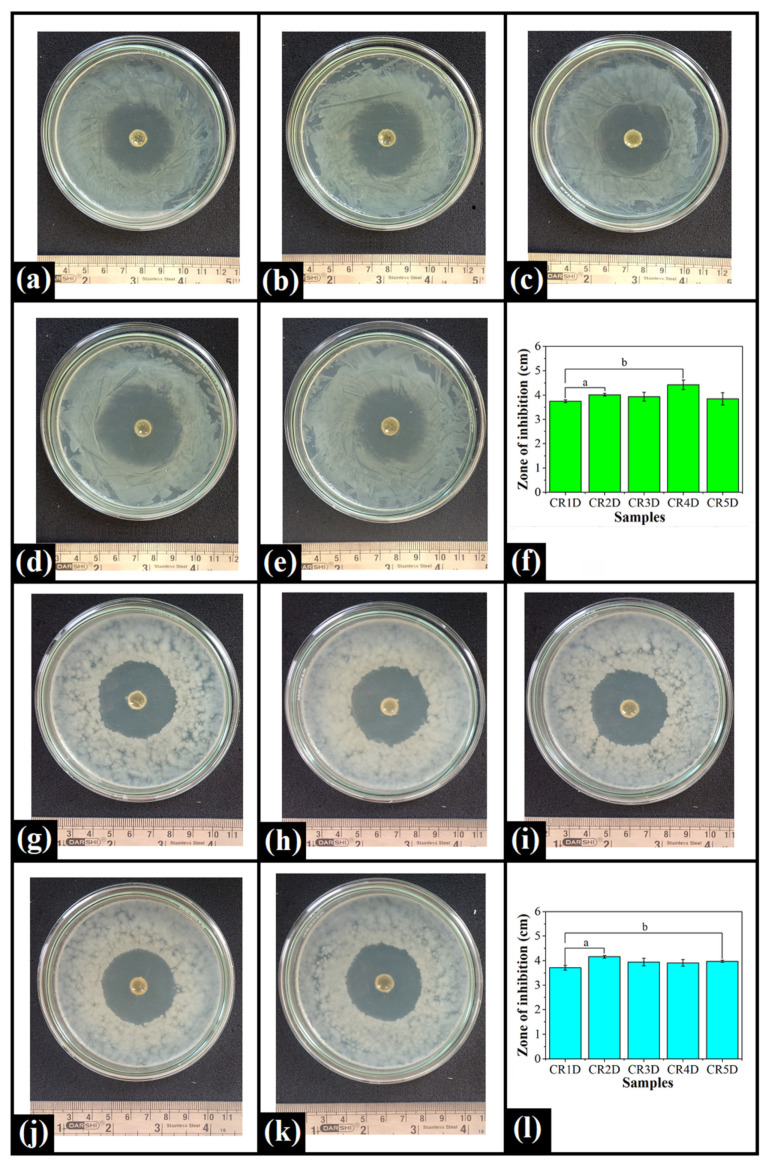
Antimicrobial activity of the prepared oleogels against *E. coli*: (**a**) CR1D, (**b**) CR2D, (**c**) CR3D, (**d**) CR4D, (**e**) CR5D, (**f**) ZOI (mean ± standard deviation); and *B. cereus*: (**g**) CR1D, (**h**) CR2D, (**i**) CR3D, (**j**) CR4D, (**k**) CR5D, (**l**) ZOI (mean ± standard deviation). Different lowercase letters denote significant differences at *p* < 0.05 level.

CPH is one of the commonly available fluoroquinolone antibiotics, which has potential bactericidal activity against a wide range of Gram-negative as well as Gram-positive bacteria [82]. In Gram-negative bacteria, CPH binds to the topoisomerase-DNA complex and forms a tripartite complex. This complex stabilizes the cleaved DNA by covalently attaching it to the ParC or GyrA subunits of topoisomerase. This is also called the “cleaved complex.” This way of stabilization of the cleaved double-strand DNA leads to lethal DNA damage, thus, killing the Gram-negative bacteria [83]. In Gram-positive bacteria, CPH inhibits bacterial type II topoisomerase IV and DNA gyrase. Topoisomerase IV is the enzyme that separates the daughter DNA strand during the bacterial cell division, while DNA gyrase is the enzyme that helps in transcription, reparation, and replication of the bacterial DNA [84]. The antibacterial action of the pristine and CPH-loaded oleogels was assessed against two model organisms namely *Escherichia coli* and *Bacillus cereus*. The pristine oleogels (without CPH; negative control group) had no antimicrobial effect against any of the target organisms (Figure S4). In contrast, CPH-loaded oleogels demonstrated excellent inhibition of *E. coli* growth, which resulted in the formation of a clear zone of inhibition (ZOI) around the well (Figure 9a–e). It can be observed that the ZOI diameter of control (CR1D), CR3D, and CR5D was similar (*p* > 0.05) (Figure 9f). On the other hand, CR2D and CR4D displayed a significantly large ZOI against *E. coli* as compared to CR1D (*p* < 0.05). Although, the variations in the ZOI diameter of CR2D, CR3D, and CR5D were statistically insignificant (*p* > 0.05), these formulations demonstrated a small ZOI diameter in contrast to CR4D (*p* < 0.05). The differences in the diameter of the ZOI can be associated with the amount of the drug released. Hence, the largest ZOI diameter of CR4D can be correlated to the observations of in vitro drug release analysis, which also demonstrated that the Fickian-mediated release of CPH was dominant in CR4D. Similar to *E. coli*, the CPH-loaded oleogels also showed clear inhibition zones against the lawns of *B. cereus* grown on the agar plates (Figure 9g–k). The ZOI diameter of CR1D was similar to CR3D and CR4D (*p* > 0.05) (Figure 9l). However, the ZOI diameter of CR1D was significantly smaller as compared to CR2D and CR5D (*p* < 0.05). CR2D showed the largest ZOI diameter and hence, the highest antibacterial activity among all the oleogels (*p* < 0.05). Additionally, the ZOI diameter shown by CR2D, CR3D, and CR4D oleogels was similar (*p* > 0.05).

### 2.9. Ocular Irritation Test

**Figure 10:**
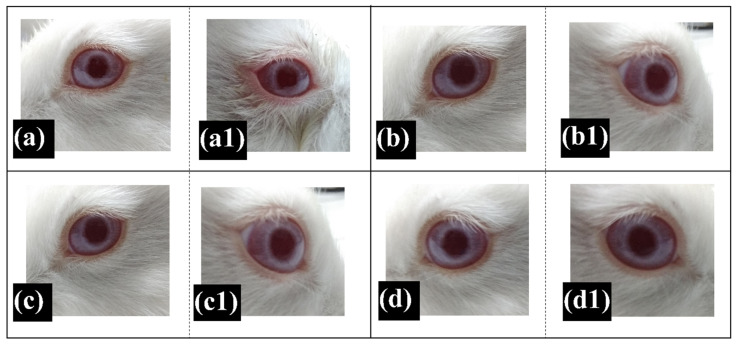
Ocular irritation study as per Draize’s rabbit eye test using CR5D. (**a**–**d**). Control eye after 1, 24, 48, and 72 h; and (**a1**,**b1**,**c1**,**d1**). Test eye after 1, 24, 48, and 72 h.

The ocular irritation study was performed according to Draize’s rabbit eye irritation test protocol. The left eye, where normal saline was administered, was used as the control. The prepared oleogels were administered within the right eye. The health conditions of both the eyes were examined at specific time intervals (1, 24, 48, and 72 h) for conjunctival redness, corneal opacity, and chemosis. Upon careful examination, we observed that there were no signs of conjunctival redness, cloudiness, chemosis, or any form of secretions from the eyes (Figure 10, Figures S5–S8). These observations persisted throughout the experiment. Hence, it can be inferred that the oleogels were well-tolerated over the surface of the eye [85]. Both the eyes (control and test) showed similar biological responses. Therefore, we may conclude that the prepared oleogels could be used for the ocular delivery of the drugs to the internal structures of the eyes.

## 3. Materials and Methods

### 3.1. Materials

CB was purchased from NV Organics (P) Ltd., New Delhi, India. RBO was procured from Marico Ltd., India. GO was prepared using the modified Hummer’s method [86]. CPH (molecular weight: 367.805 g/mol) was accepted as a gift from Aristo Pharmaceuticals Ltd., India. Nutrient agar and dialysis membrane (MW cut-off: 60 kDa) were purchased from HiMedia Pvt. Ltd., India. Freshly excised goat cornea was collected from a local licensed butcher’s house.

### 3.2. Preparation of the Oleogels

Initially, the critical gelation concentration (CGC) of CB to induce gelation of RBO was determined by heating RBO and CB at 45 °C (20 min) in a water bath, followed by homogenization using a probe sonicator (30 min). The fat solutions were then cooled at 5 °C for 30 min to induce gelation. The CGC was found by an inverted test-tube method. The CGC of CB to induce gelation of RBO was 35% (*w*/*w*). Subsequently, GO-containing oleogels were prepared by incorporating GO within the oleogel at CGC concentration. In gist, RBO, CB, and GO heated, followed by homogenization. The rest of the process remained the same. The drug-loaded oleogels were prepared by dispersing CPH into the liquid mixtures of CB/RBO/GO during the heating stage. The concentration of the drug was 0.5% (*w*/*w*) in each of the formulations. The compositions of the formulations have been tabulated in Table 5.

### 3.3. Characterization of the Oleogels

The microstructure of the oleogels was analyzed using an upright optical microscope (Leica Microsystems, model: DM750, GmbH, Berlin, Germany).

Fourier transform infrared (FTIR) spectroscopy analysis of the oleogels was carried out in the wavenumber range of 4000–600 cm^−1^ using an ATR module fitted with ZnSe crystal (Alpha-E; Bruker, Billerica, MA, USA). The recordings were carried out at a resolution of 4 cm^−1^. An average of 24 scans was accepted for the recording and the air spectrum was used as the reference.

The diffraction study of the oleogels was carried out using an X-ray diffractometer (Model: D8 Advance; Bruker, USA) in the diffraction angle range of 5–50° 2θ. The diffraction spectra were recorded at the scan rate of 5° 2θ min^−1^.

Stress relaxation (SR) analysis of the oleogels was performed using a static mechanical tester (Texture analyzer HD plus; Stable Microsystems, Godalming, UK) to assess their viscoelastic properties. The analysis was carried out using a 45° conical fixture. The testing probe entered into the oleogel sample up to a distance of 5 mm after a trigger force of 5 g. The probe was maintained at the same position for 60 s, and the corresponding variations in the force values were recorded. The speed of the probe was maintained at 1 mm s^−1^ throughout the study.

Isothermal crystallization kinetics study of the oleogels was performed using an in-house developed system. For the analysis, 10 g of the oleogel formulations were taken in glass bottles and subsequently heated at 45 °C for 15 min. The oleogel-containing glass bottles were then connected to the device and incubated in a cooling chamber (5 °C). The analysis was carried out for 1 h, and the variation in the temperature of the oleogels was recorded with respect to time.

The thermal analysis of the oleogels was performed using a differential scanning calorimeter (DSC 200 F3, Maia, Netzsch, Selb, Germany). The thermal scanning was carried out in the temperature range of 5 and 60 °C under an inert atmosphere of nitrogen at a thermal scanning rate of 2 °C min^−1^.

### 3.4. In Vitro Drug Diffusion Study

The drug release profile of CPH from the oleogels was studied for 180 min in Franz’s diffusion cell. A dialysis membrane (used as the semi-permeable membrane) was mounted between the donor and the receptor compartments. 0.5 g of the oleogel formulation was placed in the donor compartment. The receptor chamber contained 12 mL of simulated tear fluid (STF), which was maintained under continuous stirring (300 rpm) at 37 °C. The area of diffusion was 0.63 cm^2^. At regular time intervals, 1 mL of the receptor fluid was withdrawn and subsequently replaced with fresh STF. The withdrawn receptor fluid samples were spectrophotometrically analyzed at a wavelength of 277 nm using a UV-visible spectrophotometer (UV-1700, Shimadzu Corporation, Oita, Japan), and the cumulative percentage drug release was determined.

### 3.5. Biological Studies

The ex vivo permeation of CPH molecules from the oleogels was evaluated using freshly excised goat cornea. The experimental setup and procedure of the analysis were akin to that of in vitro drug release study. One major exception was the use of goat cornea as the semi-permeable membrane in place of the dialysis tubing.

The antimicrobial activity of the CPH-loaded oleogels against two model microbes was studied using the agar diffusion method. Nutrient agar plates were prepared by pouring the nutrient agar (20 mL) into sterile Petri dishes, which were then allowed to solidify. An aliquot (100 µL) of the overnight grown cultures (1 × 10^9^ CFU/mL) of *Escherichia coli* (Gram-negative) and *Bacillus cereus* (Gram-positive) were uniformly spread over each of the nutrient agar Petri dishes using a sterile glass spreader. A bore (diameter—10 mm; height—2 mm) was made at the center of each nutrient agar plate. Next, 100 µL of the CPH-loaded oleogels (molten state) was carefully poured into the well. The agar plates of both *E. coli* and *B. cereus* were then incubated at 37 °C for 12 h. The susceptibility of the model organisms against the oleogels was assessed by observing a “clear zone” around the well, which is regarded as the zone of inhibition (ZOI). After incubation of the plates, the antibacterial activity of the prepared oleogels was evaluated by measuring the diameter of the ZOI. The oleogels without the drug were considered the negative control. The antimicrobial tests were conducted in triplicate.

An in vivo ocular irritation test was performed as per the Draize’s rabbit eye test protocol [85]. For this purpose, 0.1 g of the prepared oleogel was placed within the conjunctival sac of the right eye of an albino rabbit; 0.1 mL of normal saline solution was administered in the left eye, which served as the negative control. At specific time intervals, the conjunctival sac of both the eyes was analyzed for any injuries and/or biological reactions. In our study, the experiment was carried out for 72 h. The animal study was conducted with the permission of the Institutional Animal Ethical Clearance (permission number 36/IAEC-IPT/18, dated 9/5/2018).

### 3.6. Statistical Analysis

The results of the analyses have been reported as mean ± standard deviation. *t*-test was performed using Microsoft excel software to check for significant differences at *p* < 0.05 level.

## 4. Conclusions

In this work, we developed CB/RBO/GO-based composite oleogels for controlled drug delivery of antibiotics to the internal structures of the eye. A series of formulations were prepared by incorporating varying concentrations of GO (0.005–0.05 wt%). The whitish-yellow appearance of the CB/RBO oleogels acquired a dark hue with the inclusion of GO. Microscopic studies demonstrated that the homogenous matrices of the oleogels were dispersed with globular CB crystals. A decrement in the size of the globular particles was observed with the incorporation of GO, which suggested that the growth of the fat particles was dependent on GO concentration. The FTIR spectra of the oleogels showed characteristic vibrational signals of CB and RBO. However, the molecular interactions among the oleogel components could not be established owing to the presence of GO in low concentrations. Then, the XRD analysis showed that the addition of GO enhanced the compactness and crystallite size of the CB fat crystals. The incorporation of GO within the oleogels improved the mechanical stability of the oleogels. Crystallization kinetics indicated that the incorporation of GO decreased the rate of CB crystallization in composite oleogels in a concentration-dependent manner. From both analyses, XRD and DSC, we found that the addition of GO in different concentrations altered the compactness of the CB fat crystals through the formation of β and β’ polymorphs. The in vitro release studies suggested that the release of CPH from the prepared oleogels followed either Fickian or non-Fickian diffusion or a combination of both. Additionally, the permeation of the drug molecules across the excised goat cornea was lesser than the diffusion of the drug through the dialysis membrane. This was because the cornea hindered the diffusion of the drug molecules. The agar diffusion assay demonstrated that CPH-loaded oleogels excellently inhibited the growth of both *E. coli* and *B. cereus*, which suggested that CPH retained its antimicrobial property even after its incorporation in the prepared oleogels. Furthermore, the prepared ocular formulations were found to be well-tolerated over the surface of the rabbit eyes and did not result in any kind of corneal damage/infection in the eyes of the rabbits. In summary, the prepared oleogels can be used for the controlled delivery of the drugs within the internal structure of the eyes.

## Figures and Tables

**Table 1:** XRD parameters calculated after diffraction peak deconvolution.

Formulations	Peaks	Peak Position (°2θ)	FWHM (°2θ)	d-Spacing (Å)	Crystallite Size (nm)	Lattice Strain
CR1	Peak 3	24.201	1.238	4.267	7.960	0.025
	Peak 4	25.269	0.585	4.089	16.890	0.011
	Peak 5	25.979	4.541	3.979	2.180	0.086
Average			2.122	4.112	9.010	0.041
CR2	Peak 3	21.222	4.565	4.857	2.150	0.106
	Peak 4	22.564	2.670	4.572	3.680	0.058
	Peak 5	23.743	1.166	4.348	8.450	0.024
Average			2.800	4.592	4.760	0.063
CR3	Peak 3	22.253	6.775	4.635	1.450	0.150
	Peak 4	22.253	1.978	4.635	4.970	0.044
	Peak 5	23.441	0.930	4.403	10.590	0.020
Average			3.228	4.558	5.670	0.071
CR4	Peak 3	22.659	5.259	4.553	1.870	0.115
	Peak 4	23.598	0.710	4.375	13.870	0.015
	Peak 5	24.700	0.679	4.182	14.530	0.014
Average			2.216	4.370	10.090	0.048
CR5	Peak 3	23.341	4.670	4.422	2.110	0.099
	Peak 4	24.046	0.679	4.294	14.520	0.014
	Peak 5	25.139	0.559	4.110	17.670	0.011
Average			1.969	4.275	11.433	0.041

**Table 2:** Exponential decay model parameters.

Parameters	Formulations				
	CR1	CR2	CR3	CR4	CR5
**a**	35.000	35.000	35.000	35.000	35.000
***k* (°C/m·s)**	−0.95	−0.82	−0.74	−0.89	−0.87
**R^2^**	0.995	0.998	0.998	0.994	0.998

**Table 3:** Position of endothermic signals in differential scanning calorimetry (DSC) thermograms of the prepared oleogels.

Peaks	Peak Position (Temperature, °C)				
	CR1	CR2	CR3	CR4	CR5
Peak 1 (Red arrow)	19.71	21.27	21.28	19.73	20.75
Peak 2 (green arrow)	26.84	27.36	27.86	26.35	27.36
Peak 3 (Major peak)	30.88	30.88	30.89	30.89	30.38

**Table 4:** Model parameters of in vitro drug release and ex vivo corneal permeation analysis.

Study	Model	Parameters	CR1D	CR2D	CR3D	CR4D	CR5D
Diffusion	Korsmeyer-Peppas	*K*	0.064	0.127	0.167	0.076	0.142
		*n*	0.537	0.391	0.337	0.401	0.271
		R^2^	0.994	0.999	0.999	0.994	0.997
	Peppas-Sahlin	*K_d_*	0.001	0.068	0.001	0.073	0.076
		*K_r_*	0.064	0.070	0.165	0.013	0.073
		*m*	0.268	0.228	0.169	0.293	0.165
		*K_d_*/*K_r_*	0.016	0.975	0.004	5.474	1.045
		R^2^	0.994	0.999	0.999	0.995	0.998
Corneal permeation	Korsmeyer-Peppas	*K*	0.086	0.159	0.108	0.402	0.362
		*n*	0.353	0.241	0.368	0.125	0.144
		R^2^	0.998	0.998	0.999	0.999	0.999
	Peppas-Sahlin	*K_d_*	0.062	0.128	0.007	0.394	0.270
		*K_r_*	0.032	0.038	0.101	0.0002	0.097
		*m*	0.226	0.176	0.188	0.130	0.107
		*K_d_/K_r_*	1.904	3.334	0.066	1350.906	2.779
		R^2^	0.999	0.998	0.999	0.999	0.999

**Table 5:** Composition of the prepared oleogels.

Formulations	CB (g)	RBO (g)	GO		CPH (mg)
			(mg)	(wt%)	
CR1	3.5	6.5	0.0	0.000	00
CR2	3.5	6.5	0.5	0.005	00
CR3	3.5	6.5	1.5	0.015	00
CR4	3.5	6.5	2.5	0.025	00
CR5	3.5	6.5	5.0	0.050	00
CR1D	3.5	6.5	0.0	0.000	50
CR2D	3.5	6.5	0.5	0.005	50
CR3D	3.5	6.5	1.5	0.015	50
CR4D	3.5	6.5	2.5	0.025	50
CR5D	3.5	6.5	5.0	0.050	50

## Supplementary Materials

The following are available online at https://www.mdpi.com/2310-2861/6/4/43/s1, Figure S1: Deconvoluted XRD diffractogram of the oleogels: (a) CR1, (b) CR2, (c) CR3, (d) CR4, and (e) CR5. Table S1: Parameters of the deconvoluted XRD diffractograms. Figure S2: Crystallization profiles of the prepared oleogels fitted with exponential decay model. Figure S3: Deconvoluted DSC thermograms of the prepared oleogels: (a) CR1, (b) CR2, (c) CR3, (d) CR4, and (e) CR5. Table S2: Stress relaxation model parameters. Figure S4: Antimicrobial analysis using *E. coli*: (a) CR1, (b) CR2, (c) CR3, (d) CR4, and (e) CR5; and *Bacillus cereus*: (g) CR1, (h) CR2, (i) CR3, (j) CR4, and (k) CR5. Figure S5: Ocular irritation study as per Draize’s rabbit eye test using CR1D. Figure S6: Ocular irritation study as per Draize’s rabbit eye test using CR2D, Figure S7: Ocular irritation study as per Draize’s rabbit eye test using CR3D, and Figure S8: Ocular irritation study as per Draize’s rabbit eye test using CR4D.
