# Supplementary material for: Graphene Oxide Increases Corneal Permeation of Ciprofloxacin Hydrochloride from Oleogels: A Study with Cocoa Butter-Based Oleogels
Source: Gels. 2020 Nov 23;6(4):43. doi: 10.3390/gels6040043 (PMC7709633; doi:10.3390/gels6040043)
Supplement: Supplementary file 1 [file gels-06-00043-s001.zip › gels-972203-supplementary.docx]

***Supplementary file***

**(1) XRD analysis**


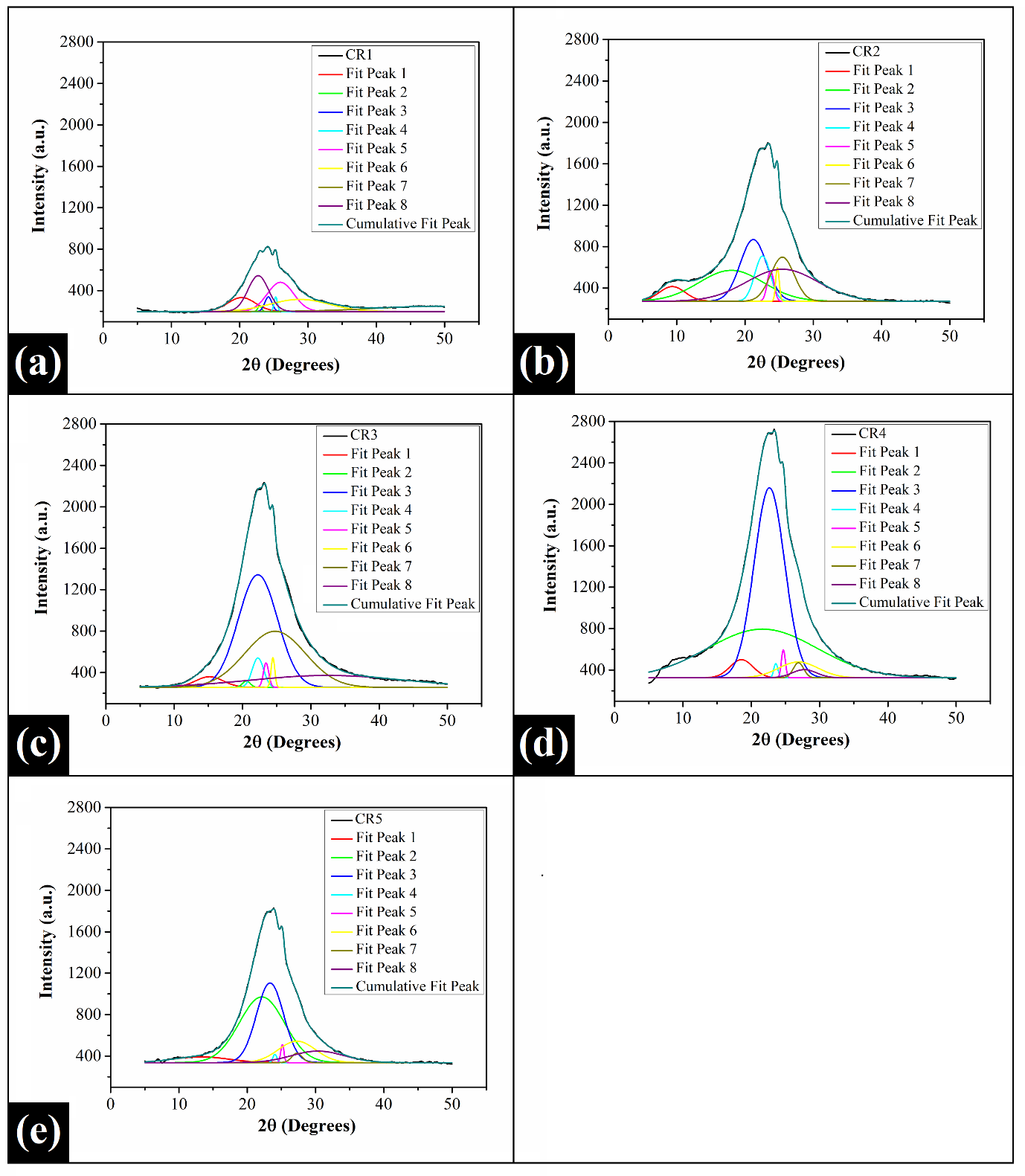


**Figure S1.** Deconvoluted XRD diffractogram of the oleogels: (a) CR1, (b) CR2, (c) CR3, (d) CR4 and (e) CR5

**Table S1.** Parameters of the deconvoluted XRD diffractograms

| **Formulations** | **Peaks** | **Peak position (^o^2θ)** | **FWHM** | **d-spacing (Å)** | **Crystallite size (nm)** | **Lattice strain** |
| --- | --- | --- | --- | --- | --- | --- |
|  | **Peak 1** | 20.289 | 4.749 | 5.079 | 2.060 | 0.116 |
|  | **Peak 2** | 22.929 | 0.636 | 4.500 | 15.470 | 0.014 |
|  | **Peak 3** | 24.201 | 1.238 | 4.267 | 7.960 | 0.025 |
| **CR1** | **Peak 4** | 25.269 | 0.585 | 4.089 | 16.880 | 0.011 |
|  | **Peak 5** | 25.979 | 4.541 | 3.979 | 2.180 | 0.086 |
|  | **Peak 6** | 28.800 | 10.152 | 3.597 | 0.980 | 0.173 |
|  | **Peak 7** | 48.829 | 21.151 | 2.164 | 0.500 | 0.203 |
|  | **Peak 8** | 22.714 | 3.547 | 4.542 | 2.770 | 0.077 |
|  | **Peak 1** | 9.360 | 4.446 | 10.963 | 2.180 | 0.237 |
|  | **Peak 2** | 18.000 | 11.100 | 5.718 | 0.880 | 0.306 |
|  | **Peak 3** | 21.222 | 4.565 | 4.858 | 2.150 | 0.106 |
| **CR2** | **Peak 4** | 22.564 | 2.670 | 4.572 | 3.680 | 0.058 |
|  | **Peak 5** | 23.743 | 1.166 | 4.348 | 8.450 | 0.024 |
|  | **Peak 6** | 24.752 | 0.608 | 4.174 | 16.230 | 0.012 |
|  | **Peak 7** | 25.477 | 4.038 | 4.057 | 2.450 | 0.078 |
|  | **Peak 8** | 25.477 | 12.271 | 4.057 | 0.810 | 0.237 |
|  | **Peak 1** | 15.156 | 4.814 | 6.783 | 2.020 | 0.158 |
|  | **Peak 2** | 20.564 | 1.361 | 5.011 | 7.200 | 0.033 |
|  | **Peak 3** | 22.253 | 6.775 | 4.635 | 1.450 | 0.150 |
| **CR3** | **Peak 4** | 22.253 | 1.978 | 4.635 | 4.970 | 0.044 |
|  | **Peak 5** | 23.441 | 0.930 | 4.403 | 10.590 | 0.020 |
|  | **Peak 6** | 24.448 | 0.650 | 4.225 | 15.190 | 0.013 |
|  | **Peak 7** | 24.774 | 10.462 | 4.170 | 0.940 | 0.208 |
|  | **Peak 8** | 32.455 | 26.455 | 3.201 | 0.380 | 0.397 |
|  | **Peak 1** | 18.576 | 3.965 | 5.542 | 2.460 | 0.106 |
|  | **Peak 2** | 21.642 | 18.885 | 4.764 | 0.520 | 0.431 |
|  | **Peak 3** | 22.659 | 5.259 | 4.553 | 1.870 | 0.115 |
| **CR4** | **Peak 4** | 23.598 | 0.710 | 4.374 | 13.870 | 0.015 |
|  | **Peak 5** | 24.700 | 0.679 | 4.182 | 14.530 | 0.014 |
|  | **Peak 6** | 26.910 | 6.885 | 3.844 | 1.440 | 0.126 |
|  | **Peak 7** | 26.910 | 1.800 | 3.844 | 5.510 | 0.033 |
|  | **Peak 8** | 27.780 | 4.192 | 3.726 | 2.370 | 0.074 |
|  | **Peak 1** | 13.187 | 9.615 | 7.790 | 1.010 | 0.363 |
|  | **Peak 2** | 22.143 | 7.789 | 4.658 | 1.260 | 0.174 |
|  | **Peak 3** | 23.341 | 4.670 | 4.422 | 2.110 | 0.099 |
| **CR5** | **Peak 4** | 24.046 | 0.679 | 4.294 | 14.510 | 0.014 |
|  | **Peak 5** | 25.139 | 0.559 | 4.110 | 17.680 | 0.011 |
|  | **Peak 6** | 27.287 | 6.415 | 3.792 | 1.550 | 0.115 |
|  | **Peak 7** | 27.287 | 1.812 | 3.792 | 5.470 | 0.033 |
|  | **Peak 8** | 30.350 | 9.094 | 3.417 | 1.100 | 0.146 |

**(2) Crystallization kinetics**


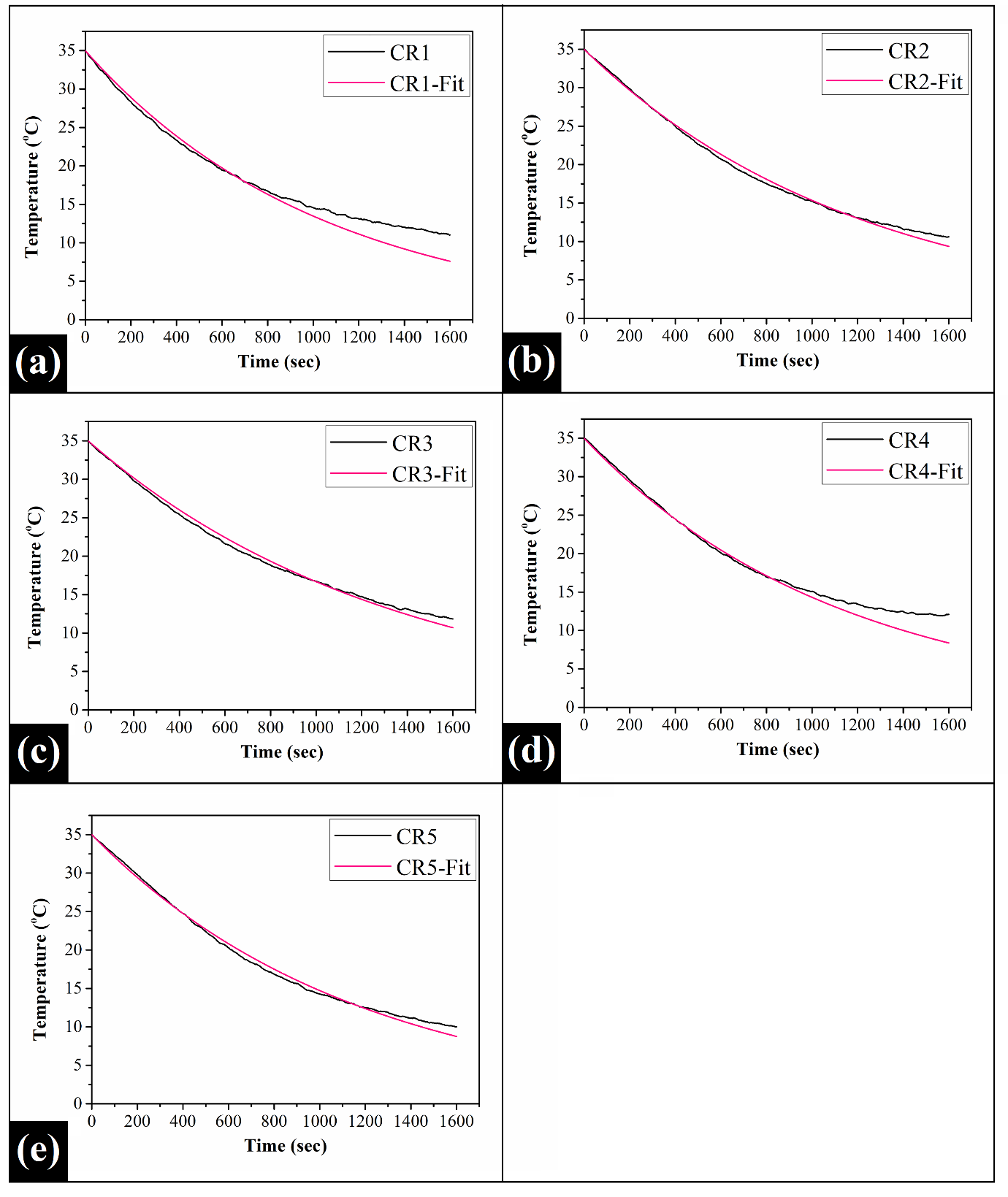


**Figure S2.** Crystallization profiles of the prepared oleogels fitted with exponential decay model.

**(3) DSC analysis**

~~
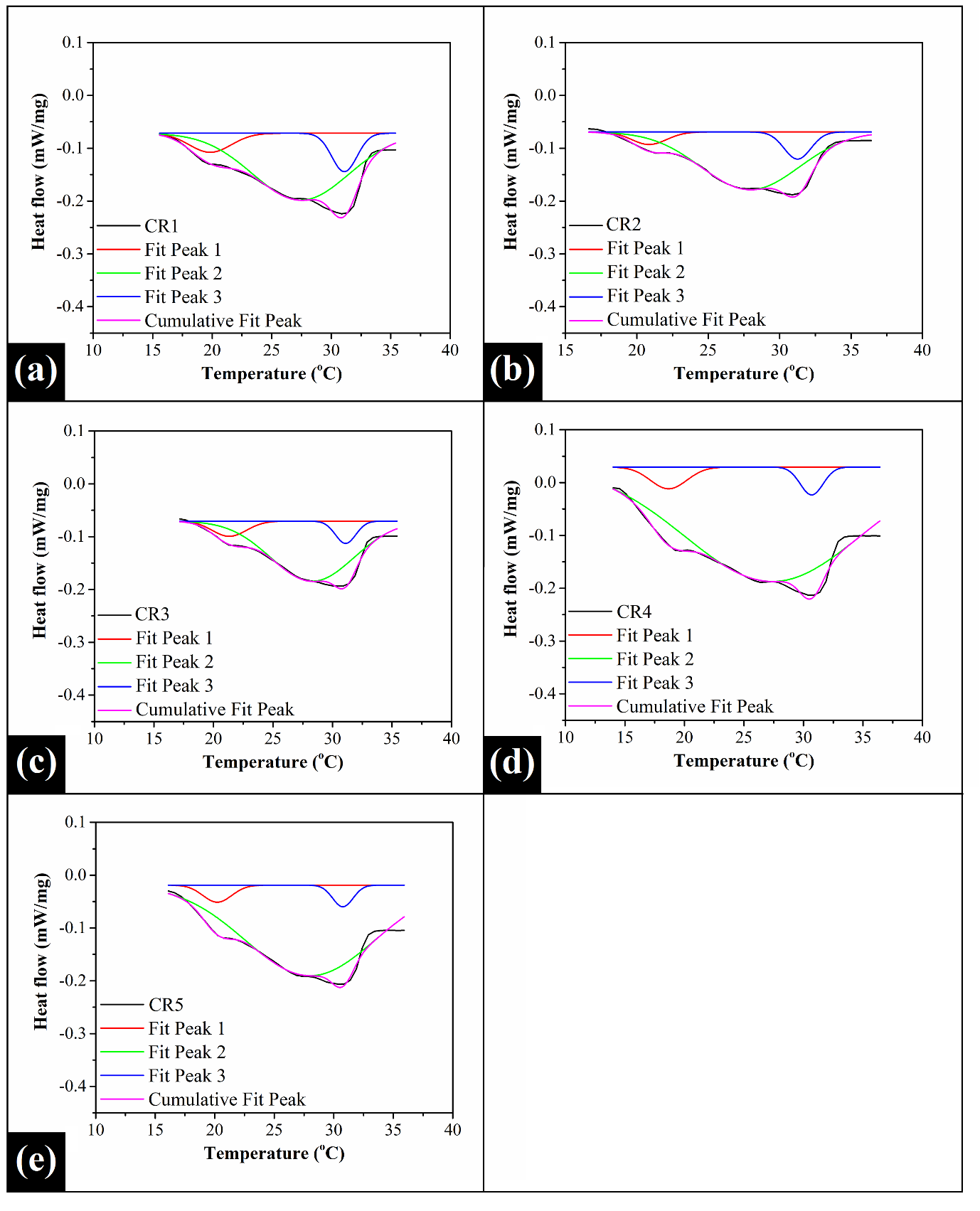
~~**Figure S3.** Deconvoluted DSC thermograms of the prepared oleogels: (a) CR1, (b) CR2, (c) CR3, (d) CR4, and (e) CR5.

**(4) Mechanical Analysis**

**Table S2. Stress relaxation model parameters**

| **Model** | **Model parameters** | **Formulations** | | | | |
| --- | --- | --- | --- | --- | --- | --- |
|  |  | **CR1** | **CR2** | **CR3** | **CR4** | **CR5** |
| **--** | **F_o_ (g)** | 745.545 ± 81.710^a^ | 753.831 ± 76.540^a^ | 1636.552 ± 483.351^b^ | 2069.221 ± 515.664^bc^ | 2829.952 ± 612.107^c^ |
|  | **F_60_ (g)** | 132.694 ± 16.181^a^ | 122.874 ± 17.255^a^ | 366.720 ± 134.880^b^ | 395.869 ± 109.236^b^ | 693.765 ± 168.201^c^ |
|  | **%SR** | 82.205 ± 0.733^a^ | 83.741 ± 0.814^ab^ | 77.874 ± 1.794^c^ | 80.963 ± 0.571^ad^ | 75.586 ± 1.005^ce^ |
| **Peleg's Model** | **k_1_** | 1.455 | 0.685 | 0.925 | 2.060 | 2.254 |
|  | **k_2_** | 1.216 | 1.206 | 1.296 | 1.194 | 1.260 |
|  | **R^2^** | 0.975 | 0.978 | 0.984 | 0.971 | 0.970 |
| **Weichert Model** | **P_0_** | 0.185 | 0.168 | 0.229 | 0.198 | 0.252 |
|  | **P_1_** | 0.251 | 0.181 | 0.222 | 0.246 | 0.194 |
|  | **τ_1_ (sec)** | 13.021 | 12.339 | 9.642 | 12.325 | 14.193 |
|  | **P_2_** | 0.562 | 0.650 | 0.548 | 0.554 | 0.554 |
|  | **τ_2_ (sec)** | 0.454 | 0.335 | 0.390 | 0.429 | 0.439 |
|  | **R^2^** | 0.998 | 0.999 | 0.999 | 0.998 | 0.998 |

Data is represented as mean ± standard deviation. Different small letters in the same row indicate significant differences using t-test (*p* < 0.05).

**(5) Antimicrobial analysis**


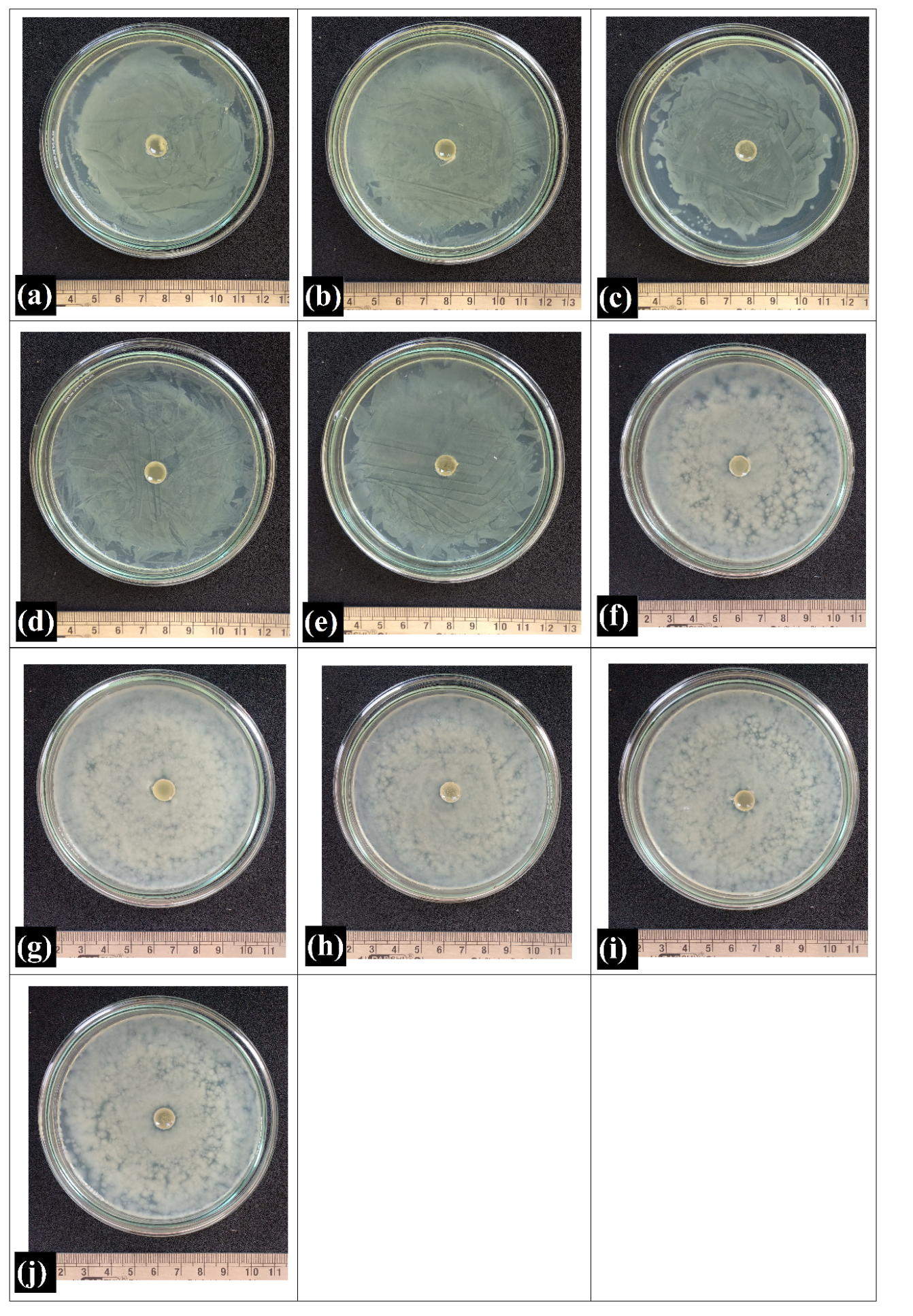


**Figure S4.** Antimicrobial analysis using *E. coli*: (a) CR1, (b) CR2, (c) CR3, (d) CR4, and (e) CR5; and *Bacillus cereus*: (g) CR1, (h) CR2, (i) CR3, (j) CR4, and (k) CR5.

**(6)** **Ocular Irritation Test**


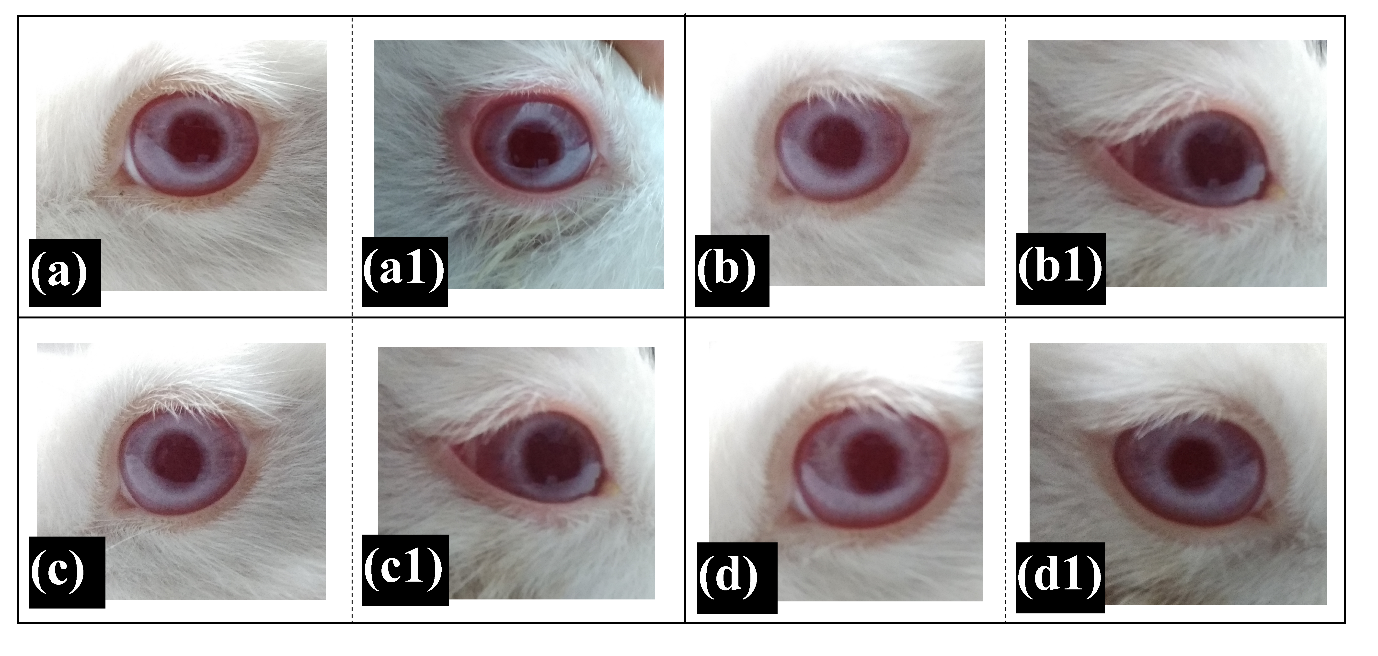


**Figure S5.** Ocular irritation study as per Draize’s rabbit eye test using CR1D.


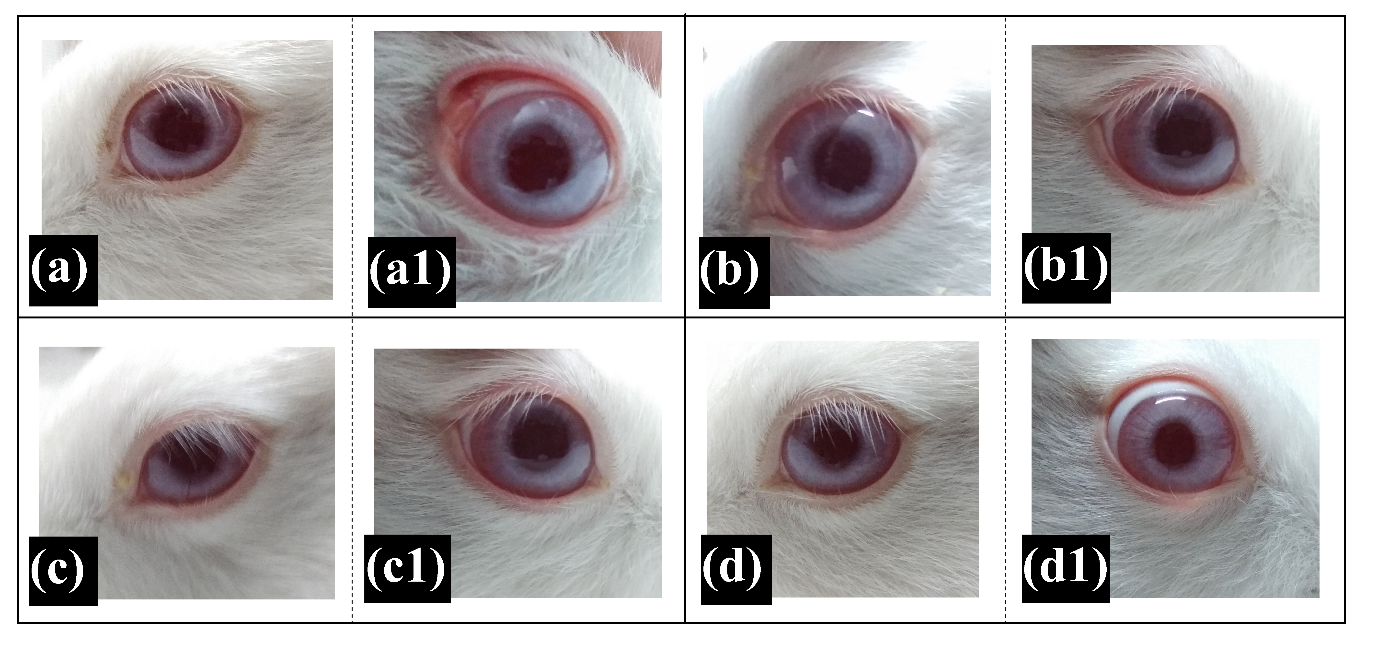
**Figure S6.** Ocular irritation study as per Draize’s rabbit eye test using CR2D.


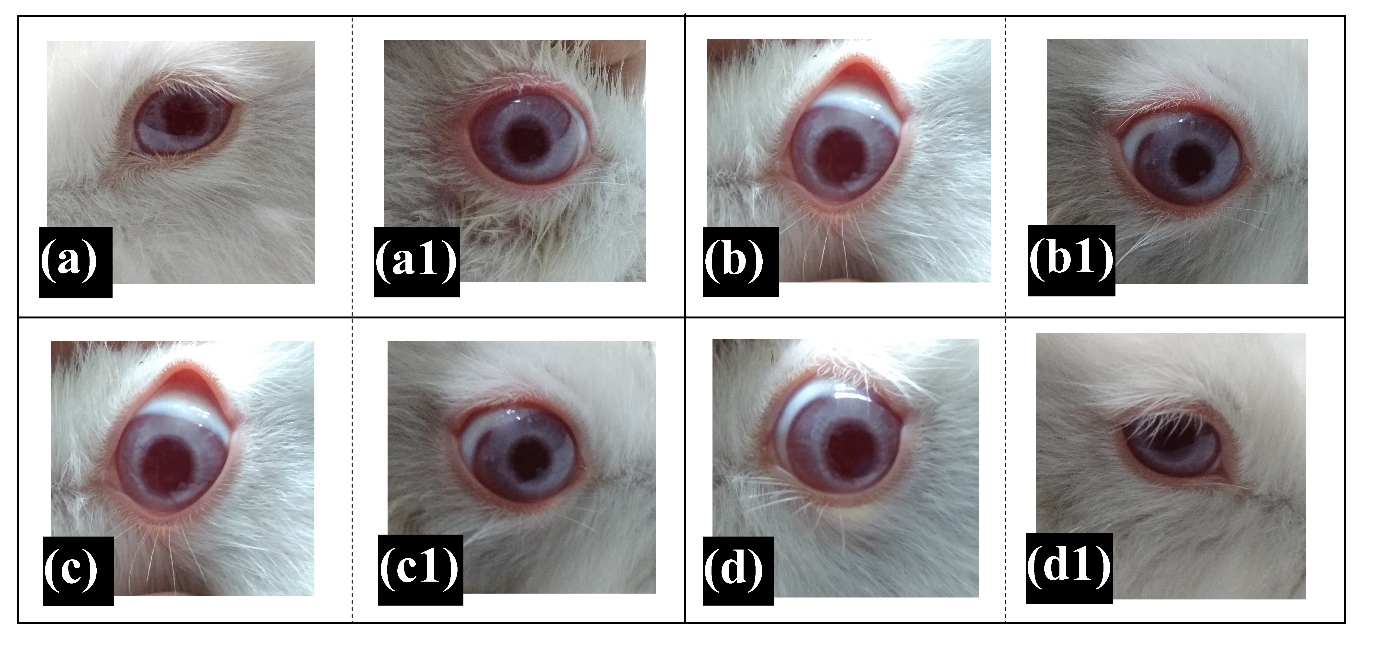
**Figure S7.** Ocular irritation study as per Draize’s rabbit eye test using CR3D.


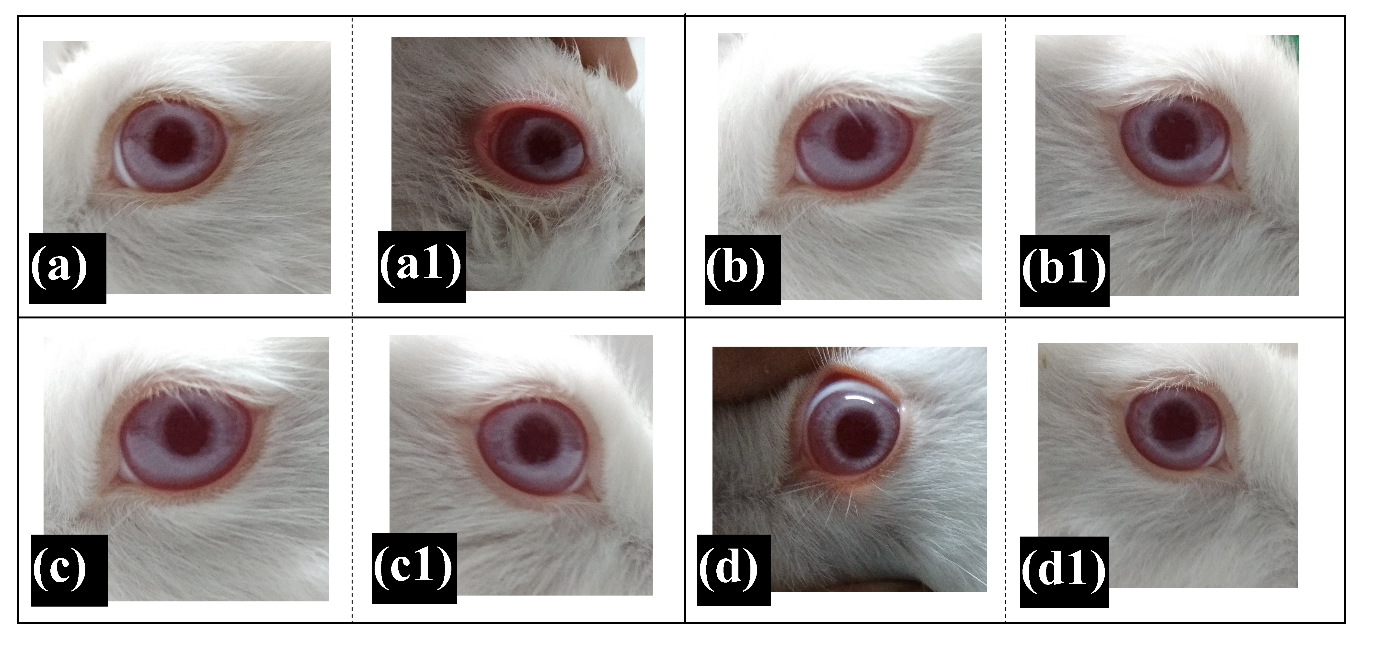
**Figure S8.** Ocular irritation study as per Draize’s rabbit eye test using CR4D.
